# Dynamics, Noise, Delays and the Gibbs and Conditional Entropy

**DOI:** 10.3390/e28040449

**Published:** 2026-04-15

**Authors:** Michael C. Mackey, Marta Tyran-Kamińska

**Affiliations:** 1 Departments of Physiology, Physics & Mathematics, McGill University, 3655 Promenade Sir William Osler, Montreal, QC H3G 1Y6, Canada; 2 Institute of Mathematics, University of Silesia in Katowice, Bankowa 14, 40-007 Katowice, Poland; mtyran@us.edu.pl

**Keywords:** density evolution, Ornstein–Uhlenbeck process, Gaussian process, delay differential equation, stochastic functional differential equations

## Abstract

In this paper we review the concepts of the Gibbs and conditional entropies and examine their dynamic behaviour when the underlying dynamics are described by ordinary differential equations or stochastic differential equations. We then go on to introduce techniques for the analogous examination when the dynamics involve delays and noise. It is found that the effects of stochastic perturbations and/or delayed dynamics may be such that the approach of the entropies to equilibrium are not necessarily monotone and are dependent on system parameters.

## 1. Introduction

The inexorable passage of time both fascinates and terrifies humans: witness the amount of money spent yearly trying to cosmetically reverse, or slow down, the effects of the passage of time, to obtain an idea of just how concerning people find it. This one-way evolution of time is, of course, simply a reflection of the operation of the second law of thermodynamics. Were it not for the consequences of the second law there would presumably be far less panic about the passage of time. We are quite sure, however, there would still be a lingering curiosity about why time evolves in only one direction.

The development of thermodynamics was spurred on by developments in industrial processes during the early to mid-nineteenth century as people had to deal with the concepts of heat, work, and temperature in the pursuit of increased efficiency of steam engines. The initial work by Sadi Carnot led to the formulation of the laws of thermodynamics, due to the efforts of William Thompson (later Lord Kelvin) and Rudolf Clausius. These developments in thermodynamics, including the ‘laws of thermodynamics’, culminated in the development of statistical mechanics, which makes predictions of the collective macroscopic behaviour of particles from their microscopic behaviours.

The second law of thermodynamics states that in a closed (no transfer of matter or energy in or out) system the entropy of the system can only increase (or stay constant) as time progresses. We may characterize this as the *strong form* of the second law, while the *weak form* would simply specify that the entropy will approach zero in the limit of long times. The strong form clearly implies the weak form.

In the latter part of the nineteenth century, in the developing field of statistical mechanics, individuals started to seriously contemplate how to define and compute this quantity known as entropy. Three names of giants come to mind—Ludwig Boltzmann (Germany), Henri Poincaré (France), and J. Willard Gibbs (USA)—for their insights into the concept of entropy. For reasons that we will discuss later, Gibbs was the clear winner, and Section 2 will introduce the Gibbs entropy and a generalization of it. The remainder of the paper will deal with the issue of the temporal change in entropy, and this will be based on considerations from physics. Section 3 commences our consideration of the temporal evolution of entropy, as enshrined in the second law of thermodynamics, by reviewing entropy evolution when the underlying dynamics are defined by ordinary differential equations. We then continue in the same section to consider the added effects of noise on entropy evolution. This is a review of the material presented in [1,2].

Section 4 constitutes the bulk of this paper, adding the possibility of *delays* in dynamics to the mix of entropy evolution and the second law. In Section 4.1 we briefly review multidimensional stochastic functional differential equations, and in Section 4.2 we turn to a consideration of general linear equations with Gaussian noise, as it is only for such equations that we know what the density evolution is and what the entropies are. This examination of the effects of Gaussian noise in linear systems as a first approximation is only possible because of the well-developed theory of Gaussian processes with continuous sample paths. We derive various formulas for entropy dynamics, and show that the conditional entropy will approach zero but need not do so as a monotone function of time. Though linear dynamics driven by Gaussian noise is well motivated by analytical tractability and by its relevance to a broad class of stochastic processes, it is essential to emphasize to the reader that the closed-form entropy expressions we obtain here rely crucially on these assumptions. In particular, the Gaussian structure and linear evolution ensure that the state remains fully characterized by its first two moments, a property that generally fails in nonlinear and/or non-Gaussian systems. Consequently, the results derived here should not be interpreted as generic entropy laws applicable beyond the linear/Gaussian framework.

In Section 4.3 we give examples of non-monotone convergence to zero of the conditional entropy. First we consider a one-dimensional example as initially presented in [3], and next we present completely new material on a second-order differential delay equation perturbed by noise. Section 4.4 contains a derivation of analytical expressions for the variances of the stationary position and velocity variables from Section 4.3. Finally in Section 4.5 we introduce one possible approach to obtain a Fokker–Planck-type equation for stochastic delay differential equations, extending the material presented in [3,4] to delay equations with both discrete and distributed delays. We conclude with some brief remarks in Section 5.

## 2. Entropies: Gibbs and Conditional

In this paper we are concerned with the dynamic behaviour of entropies that are relevant for physical systems, and thus we confine our attention to the Gibbs entropy, first considered in Gibbs [5], and the conditional entropy which generalizes the Gibbs entropy. The latter has been the object of study by a number of different authors [6,7,8].

In his original work Gibbs assumed that he was dealing with a system operating in a phase space *X* which attains a steady-state density $f_{*}$. Given this steady-state density he defined the ‘index of probability’ as $\ln f_{*}\left(x\right)$ (ln is the natural logarithm) and identified the steady-state (equilibrium) entropy to be

(1) $$H_{G}\left(f_{*}\right)=-\int_{X}f_{*}\left(x\right)\ln f_{*}\left(x\right)\;dx.$$

The equilibrium entropy definition, according to Gibbs, in (1) is the standard for equilibrium computations in thermodynamics and statistical mechanics and has led to the identification of $H_{G}\left(f_{*}\right)$ with the equilibrium thermodynamic entropy.

There is no particular reason to restrict the definition of the Gibbs entropy in (1) to equilibrium situations, and indeed we extend it to define the time-dependent Gibbs entropy of f(x,t) by

(2) $$H_{G}\left(f\left(\cdot ,t\right)\right)=-\int_{X}f\left(x,t\right)\ln f\left(x,t\right)\;dx.$$

In addition to generalizing the Gibbs entropy to time-dependent situations we can also generalize the Gibbs entropy to situations in which we have a *non-uniform steady-state density* $f_{*}$. This generalization has acquired the name of *conditional entropy* [9] and is given by

(3) $$H_{c}\left(f\left(\cdot ,t\right)|f_{*}\right)=-\int_{X}f\left(x,t\right)\ln\frac{f(x,t)}{f_{*}\left(x\right)}dx.$$

It should be noted that $-H_{c}\left(f\left(\cdot ,t\right)|f_{*}\right)$ is also known as the Kullback–Leibler divergence (I-divergence or relative entropy) [10]. It measures the distance between the two densities and it satisfies $-H_{c}\left(f\left(\cdot ,t\right)|f_{*}\right)\geq 0$.

But what about the Austrian giant Ludwig Boltzmann and his original entropy [11] (actually there were several different varieties of Boltzmann entropies and [12] has untangled and clarified their inter-relationships)? Problems with the Boltzmann entropy have been very elegantly and clearly analyzed in [13]. The Boltzmann entropy fails to give proper equilibrium entropies in several physical situations [14], while the Gibbs entropy does not suffer from this defect. Secondly, the Gibbs and Boltzmann entropies are only in agreement for extremely dilute gases in which the constituents do not interact. Finally, studies [2] have identified situations in which the Boltzmann entropy behaves non-physically but the Gibbs entropy does not. There is a very nice study of Boltzmann versus Gibbs entropy [15] for the interested reader.

To conclude this section, we recall the amusing quotation from Eddington [16] re-framing the issue of temporal unidirectionality in terms of the behaviour of *entropy*, saying

“The law that entropy always increases holds, I think, the supreme position among the laws of Nature. If someone points out to you that your pet theory of the universe is in disagreement with Maxwell’s equations—then so much the worse for Maxwell’s equations. If it is found to be contradicted by observation—well, these experimentalists do bungle things sometimes. But if your theory is found to be against the Second Law of Thermodynamics I can give you no hope; there is nothing for it to collapse in deepest humiliation.”

## 3. Behavior of Entropies for Dynamics Described by Differential Equations and Their Stochastic Perturbations

### 3.1. Density Evolution Equations

Consider the dynamics defined by the system of ordinary differential equations

$$\frac{dx_{i}}{dt}=F_{i}\left(x\right),\;i=1,\ldots ,d,$$

with initial conditions $x_{i}\left(0\right)=x_{i,0}$ from a subset of $R^{d}$. We rewrite it in the vector form as

$$x^{\prime }\left(t\right)=F\left(x\left(t\right)\right),\;t>0,\;x\left(0\right)=x_{0}.$$

The probability density f(x,t), if it exists, of the variable x(t) satisfies the generalized Liouville equation

(4) $$\frac{\partial f}{\partial t}=-\sum_{i}\frac{\partial (fF_{i})}{\partial x_{i}},$$

and if there exists a steady-state density $f_{*}$, then it is the stationary solution of Equation (4):

$$\sum_{i}\frac{\partial (f_{*}F_{i})}{\partial x_{i}}=0.$$

We next turn our attention to the Itô-type stochastic differential equation

(5) $$dx\left(t\right)=F\left(x\left(t\right)\right)dt+\Sigma \left(x\left(t\right)\right)\;dw\left(t\right),\;t>0,\;x\left(0\right)=x_{0},$$

where $\Sigma \left(x\right)=[\sigma_{ij}\left(x\right)]$ is a d×n matrix and *w* is an *n*-dimensional standard Wiener process on an underlying probability space (Ω,F,P). Recall that a standard Wiener process is a Gaussian process with continuous paths and stationary independent increments for which

$$E\left(w\left(t\right)-w\left(s\right)\right)=0,\;E(\left(w\left(t\right)-w\left(s\right)\right){\left(w\left(t\right)-w\left(s\right)\right)}^{T})=I|t-s|,\;t,s\geq 0,$$

where the superscript *T* denotes the transpose and *I* is the identity matrix. Here and later E is the expectation operator corresponding to the probability measure P. We assume that both *F* and Σ satisfy a global Lipschitz condition. It is known [17,18,19] that if x(t) is the solution to the stochastic differential Equation (5) then the evolution of the density function f(x,t) of x(t) is described by the *Fokker–Planck equation* (Kolmogorov forward equation)

(6) $$\frac{\partial f}{\partial t}=-\sum_{i=1}^{d}\frac{\partial [F_{i}\left(x\right)f]}{\partial x_{i}}+\frac{1}{2}\sum_{i,j=1}^{d}\frac{\partial^{2}\left[a_{ij}\left(x\right)f\right]}{\partial x_{i}\partial x_{j}}$$

where

$$a_{ij}\left(x\right)=\sum_{k=1}^{n}\sigma_{ik}\left(x\right)\sigma_{jk}\left(x\right).$$

Moreover, if there exists a stationary density $f_{*}\left(x\right)$ then it is the solution of the stationary Fokker–Planck Equation (6):

(7) $$-\sum_{i=1}^{d}\frac{\partial [F_{i}\left(x\right)f]}{\partial x_{i}}+\frac{1}{2}\sum_{i,j=1}^{d}\frac{\partial^{2}\left[a_{ij}\left(x\right)f\right]}{\partial x_{i}\partial x_{j}}=0.$$

#### 3.1.1. Gibbs and Conditional Entropies

Differentiating with respect to time the Gibbs entropy, as given by Equation (2), leads to

$$\frac{dH_{G}}{dt}=\int f\left(\sum_{i}\frac{\partial F_{i}\left(x\right)}{\partial x_{i}}-\frac{1}{2}\sum_{i,j}\frac{\partial^{2}a_{ij}\left(x\right)}{\partial x_{i}\partial x_{j}}\right)\;dx+\frac{1}{2}\int \frac{1}{f}\sum_{i,j=1}^{d}a_{ij}\left(x\right)\frac{\partial f}{\partial x_{i}}\frac{\partial f}{\partial x_{j}}\;dx.$$

Note that if $a_{ij}$ values do not depend on *x* then we obtain

(8) $$\frac{dH_{G}}{dt}=\int f\sum_{i}\frac{\partial F_{i}\left(x\right)}{\partial x_{i}}\;dx+\frac{1}{2}\sum_{i,j=1}^{d}a_{ij}\int \frac{1}{f}\frac{\partial f}{\partial x_{i}}\frac{\partial f}{\partial x_{j}}\;dx.$$

In particular, the second term in the right-hand side of (8) is non-negative definite, but the first term is of indeterminant sign [20] (Equation (14)). Thus *the temporal behavior of the Gibbs entropy in its approach to equilibrium is unclear*.

Next, if we differentiate Equation (3) for the conditional entropy with respect to time, then we obtain

$$\frac{dH_{c}}{dt}=\frac{1}{2}\int \left(\frac{f_{*}^{2}}{f}\right)\sum_{i,j=1}^{d}a_{ij}\left(x\right)\frac{\partial }{\partial x_{i}}\left(\frac{f}{f_{*}}\right)\frac{\partial }{\partial x_{j}}\left(\frac{f}{f_{*}}\right)\;dx,$$

where we used (6), integration by parts and Equation (7) for the stationary density $f_{*}$. Consequently,

(9) $$\frac{dH_{c}}{dt}\geq 0,$$

because the matrix $\left(a_{ij}\left(x\right)\right)$ is non-negative definite. Hence, we conclude that *the conditional entropy is a monotone increasing function of time and satisfies the strong form of the second law*. In particular, if all $a_{ij}$ values are equal to zero (no noise) then $\frac{dH_{c}}{dt}=0$, implying that in the noise-free case when *f* is the solution of the Liouville Equation (4), then

(10) $$H_{c}\left(f\left(\cdot ,t\right)|f_{*}\right)\equiv H_{c}\left(f_{0}|f_{*}\right).$$

This last result deserves some consideration since it is so important.

1. All of the formulations of fundamental classical, relativistic, and quantum physics are in terms of ordinary or partial differential equations and without noise.
2. Thus any temporal evolution of entropy in these systems would appear to be impossible.
3. It could only occur that the entropy might change (see (9)) in the presence of noise.

This is astonishing and pinpoints exactly the conundrum that physics has always faced (Equation (10)) in terms of trying to find a dynamical foundation for the second law of thermodynamics (either weak or strong).

#### 3.1.2. Convergence to Zero of the Conditional Entropy

Various conditions implying convergence to zero of the conditional entropy are known; see, e.g., Mackey and Tyran-Kamińska [1] for a review. Here we only indicate how one can obtain a lower bound on the conditional entropy $H_{c}\left(f\left(\cdot ,t\right)|f_{*}\right)$ knowing that the probability density function $p(x,t|x_{0})$ of x(t) conditional on $x\left(0\right)=x_{0}$ exists. One identifies the transition probability $p(x,t|x_{0})$ as a solution of the Fokker–Planck Equation (6) with the initial condition $\delta (x-x_{0})$. Then we obtain

$$f\left(x,t\right)=\int p\left(x,t|x_{0}\right)f_{0}\left(x_{0}\right)\;dx_{0},$$

where $f_{0}$ is the density of the initial condition $x_{0}$, leading to

(11) $$0\geq H_{c}\left(f\left(\cdot ,t\right)|f_{*}\right)\geq \int H_{c}\left(p\left(\cdot ,t|x_{0}\right)|f_{*}\right)f_{0}\left(x_{0}\right)dx_{0}$$

by Jensen’s inequality (see [3]). Consequently, if $\lim_{t\to \infty }H_{c}\left(p\left(\cdot ,t|x_{0}\right)|f_{*}\right)=0$ for all $x_{0}$ then

$$\lim_{t\to \infty }H_{c}\left(f\left(\cdot ,t\right)|f_{*}\right)=0.$$

### 3.2. Effects of Noise

Specific examples for which one can determine the densities f(·,t) and $f_{*}$ and the corresponding entropies are mainly known for linear differential equations with additive noise. Our first example is an Ornstein–Uhlenbeck process that was developed as a perturbation to the velocity *v* of a Brownian particle and is given by the Langevin equation

$$\frac{dv}{dt}=-\gamma v+\sigma \xi \left(t\right),$$

where γ>0,σ>0 and ξ(t) is white noise. In this case (5) reduces to

dv(t)=−γv(t)dt+σdw(t),

where w(t) is a standard one-dimensional Wiener process. Another frequently used example is a noisy harmonic oscillator

(12) $$\frac{d^{2}}{dt^{2}}x\left(t\right)+\gamma \frac{d}{dt}x\left(t\right)+\beta x\left(t\right)=\sigma \xi \left(t\right)$$

with positive constant coefficients. If we introduce the variables

$$x\left(t\right)=\left(\begin{array}{c} x(t)\\ v(t) \end{array}\right)\;\mathrm{with}\;v\left(t\right)=x^{\prime }\left(t\right),$$

then we obtain

dx(t)=Ax(t)dt+Σdw(t),

where

$$A=\left(\begin{array}{cc} 0 & 1\\ -\beta & -\gamma \end{array}\right)\;\mathrm{and}\;\Sigma =\left(\begin{array}{c} 0\\ \sigma \end{array}\right).$$

Thus, in this section we consider the general multidimensional Ornstein–Uhlenbeck process

(13) dx(t)=Ax(t)dt+Σdw(t)

where *A* is a d×d matrix and Σ is a d×n matrix. The solution to Equation (13) is

(14) $$x\left(t\right)=e^{tA}x\left(0\right)+\int_{0}^{t}e^{(t-r)A}\Sigma \;dw\left(r\right)$$

where $e^{tA}=\sum_{k=0}^{\infty }\frac{t^{k}}{k!}A^{k}$. Note that $X\left(t\right)=e^{tA}$ is the solution to $X^{\prime }\left(t\right)=AX\left(t\right)$ satisfying X(0)=I and it is called the fundamental solution of the deterministic equation $x^{\prime }\left(t\right)=Ax\left(t\right)$. The properties of stochastic integrals imply that the process

$$\zeta \left(t\right)=\int_{0}^{t}X\left(t-s\right)\Sigma \;dw\left(r\right),\;t\geq 0,$$

is Gaussian. Its mean vector is equal to 0 and its covariance function is given by

$$E\left(\zeta \left(t\right)\zeta {\left(t+s\right)}^{T}\right)=\int_{0}^{t}e^{(t-r)A}\Sigma \Sigma^{T}e^{\left(t+s-r\right)A^{T}}dr,$$

by Itô’s isometry. In particular, the distribution of ζ(t) is Gaussian with mean 0 and the variance–covariance matrix

(15) $$R\left(t\right):=E\left(\zeta \left(t\right)\zeta {\left(t\right)}^{T}\right)=\int_{0}^{t}e^{rA}\Sigma \Sigma^{T}e^{rA^{T}}dr.$$

Observe that the matrix R(t) is symmetric. Since $e^{rA}\Sigma \Sigma^{T}e^{rA^{T}}$ is non-negative definite for all r≥0, the matrix R(t) is also nonnegative definite, but it might not be positive definite. In particular, if the Kalman rank condition holds [21,22], i.e., for the d×d matrix *A* and the d×n matrix Σ, the d×dn block matrix

$$\left[A|\Sigma \right]:=\left[\Sigma \;A\Sigma \;\ldots \;A^{d-1}\Sigma \right]$$

is of full rank (rank[A|Σ]=d), then the matrix R(t) is of full rank. Consequently, R(t) is positive definite for all t>0, or equivalently detR(t)>0.

Let $f_{m,Q}$ be a Gaussian density with mean vector *m* and positive definite covariance matrix *Q*, i.e.,

(16) $$f_{m,Q}\left(x\right)=\frac{1}{{\left(2\pi \right)}^{d/2}{\left(\det Q\right)}^{1/2}}e^{-\frac{1}{2}{\left(x-m\right)}^{T}Q^{-1}\left(x-m\right)},\;x\in R^{d},$$

where $Q^{-1}$ is the inverse of the matrix *Q*. Assume that the density $f_{0}$ of x(0) is $f_{m,Q}$. Then x(t) as given in (14) has a Gaussian distribution with mean vector $E\left(x\left(t\right)\right)=e^{tA}m$ and the covariance matrix $\bar{R}\left(t\right)$ is of the form

(17) $$\bar{R}\left(t\right)=e^{tA}Qe^{tA^{T}}+R\left(t\right),\;t>0.$$

Consequently, the distribution of x(t) does not depend on *t* if and only if m=0 and there exists a covariance matrix $R_{*}$ such that

(18) $$R_{*}=e^{tA}R_{*}e^{tA^{T}}+R\left(t\right),\;t>0.$$

Differentiating (18) with respect to *t* and then setting t=0 we obtain the following equation for $R_{*}$:

(19) $$AR_{*}+R_{*}A^{T}+\Sigma \Sigma^{T}=0.$$

Thus the covariance matrix $R_{*}$ satisfies (19). Conversely, if $R_{*}$ is the solution of (19) then (18) holds as well. Note that if R(t) is positive definite then $R_{*}$ is also positive definite by (18).

#### 3.2.1. Existence of a Stationary Density

In what follows we assume that rank[A|Σ]=d. Then x(t) with $x\left(0\right)=x_{0}$ has a Gaussian distribution with mean $e^{tA}x_{0}$ and a positive definite covariance R(t) as in (15). Letting $R{\left(t\right)}^{-1}$ be the inverse matrix of R(t), the density of x(t) is given by

$$p\left(x,t|x_{0}\right)=\frac{1}{{\left(2\pi \right)}^{d/2}{\left(\det R\left(t\right)\right)}^{1/2}}\exp\left\{-\frac{1}{2}{\left(x-e^{tA}x_{0}\right)}^{T}R{\left(t\right)}^{-1}\left(x-e^{tA}x_{0}\right)\right\}.$$

A stationary density $f_{*}$ exists if and only if the matrix *A* has only eigenvalues with negative real parts. In that case, the stationary density $f_{*}$ has the form

(20) $$f_{*}\left(x\right)=\frac{1}{{\left(2\pi \right)}^{d/2}{\left(\det R_{*}\right)}^{1/2}}\exp\left\{-\frac{1}{2}x^{T}R_{*}^{-1}x\right\},$$

with the positive definite matrix $R_{*}$ being the unique symmetric matrix satisfying (19) and given by

$$R_{*}=\int_{0}^{\infty }e^{sA}\Sigma \Sigma^{T}e^{sA^{T}}ds.$$

Since $R\left(t\right)\to R_{*}$ and $e^{tA}\to 0$ as t→∞, we have

$$\lim_{t\to \infty }p\left(x,t|x_{0}\right)=f_{*}\left(x\right).$$

Consequently, every stationary solution of the corresponding Fokker–Planck equation is a constant multiple of the stationary density $f_{*}$.

A stationary solution of (13) can be represented as

$$Z\left(s\right)=\int_{-\infty }^{s}e^{(s-r)A}\Sigma dw\left(r\right),\;s\in R,$$

where we extend the Wiener process *w* to R taking its independent copy $\left\{\bar{w}\left(t\right):t\geq 0\right\}$ and setting $w\left(r\right)=-\bar{w}\left(-r\right)$ for r<0. Observe that

$$Z\left(t\right)=e^{tA}Z\left(0\right)+\zeta \left(t\right),\;t\geq 0,$$

implying that Z(t) is a solution of (13). Since ζ(t) is a Gaussian distributed vector, we see that Z(t) has a Gaussian distribution with mean 0 and variance–covariance matrix $R_{*}$. Consequently, Z(t), t≥0, is a stationary solution of (13) and $f_{*}$ is the density of Z(t).

#### 3.2.2. Noise and the Temporal Behaviour of Gibbs and Conditional Entropies

For the Gaussian density $f_{m,Q}$ in (16), the Gibbs entropy is

(21) $$H_{G}\left(f_{m,Q}\right)=\frac{1}{2}\left(d+\ln\left[{\left(2\pi \right)}^{d}\det Q\right]\right),$$

while for two Gaussian densities $f_{m_{1},Q_{1}}$ and $f_{m_{2},Q_{2}}$ the conditional entropy is

(22) $$\begin{array}{c} H_{c}\left(f_{m_{1},Q_{1}}|f_{m_{2},Q_{2}}\right)=\frac{1}{2}\ln\frac{\det Q_{1}}{\det Q_{2}}+\frac{1}{2}\mathrm{tr}\left[\left(Q_{1}^{-1}-Q_{2}^{-1}\right)Q_{1}\right]\\ -\frac{1}{2}\mathrm{tr}\left[Q_{2}^{-1}\left(m_{1}-m_{2}\right){\left(m_{1}-m_{2}\right)}^{T}\right].\; \end{array}$$

Consequently, if $f_{0}=f_{m,Q}$ with m=E(x(0)) and $Q=E\left(x\left(0\right)-m\right){\left(x\left(0\right)-m\right)}^{T}$, then for the density f(x,t) of x(t) we obtain the following

(23) $$H_{G}\left(f\left(\cdot ,t\right)\right)=\frac{1}{2}\left(d+\ln\left[{\left(2\pi \right)}^{d}\det\bar{R}\left(t\right)\right]\right),$$

where $\bar{R}\left(t\right)$ is given by (17) and

(24) $$H_{c}\left(f\left(\cdot ,t\right)|f_{*}\right)=\frac{1}{2}\ln\frac{\det\bar{R}\left(t\right)}{\det R_{*}}+\frac{1}{2}\mathrm{tr}\left(I-R_{*}^{-1}\bar{R}\left(t\right)\right)-\frac{1}{2}\mathrm{tr}\left(R_{*}^{-1}e^{tA}mm^{T}e^{tA^{T}}\right).$$

Note that if $Q=R_{*}$ then $\bar{R}\left(t\right)=R_{*}$ and Equation (24) reduces to

$$H_{c}\left(f\left(\cdot ,t\right)|f_{*}\right)=-\frac{1}{2}\mathrm{tr}\left(R_{*}^{-1}e^{tA}mm^{T}e^{tA^{T}}\right)$$

which is always an increasing function of time when m≠0.

Observe that if $m=x\left(0\right)=:x_{0}$ and Q=0, then we recover $H_{c}\left(p\left(\cdot ,t|x_{0}\right)|f_{*}\right)$ from Equation (24). Thus, for an arbitrary initial density $f_{0}$ with finite variance, we obtain from (11) the lower bound

$$H_{c}\left(f\left(\cdot ,t\right)|f_{*}\right)\geq \frac{1}{2}\ln\frac{\det R(t)}{\det R_{*}}+\frac{1}{2}\mathrm{tr}\left(I-R_{*}^{-1}R\left(t\right)\right)-\frac{1}{2}\int \mathrm{tr}\left(R_{*}^{-1}e^{tA}x_{0}x_{0}^{T}e^{tA^{T}}\right)f_{0}\left(x_{0}\right)dx_{0}$$

for the conditional entropy. Since

$$\lim_{t\to \infty }e^{tA}=0\;\mathrm{and}\;\lim_{t\to \infty }R\left(t\right)=R_{*},$$

it is clear that $\lim_{t\to \infty }H_{c}\left(f\left(\cdot ,t\right)|f_{*}\right)=0$. Remember that we have ${\dot{H}}_{c}\left(f\left(\cdot ,t\right)|f_{*}\right)\geq 0$ by (9) so *we have the strong form of the second law obeyed in this case*.

Thus, the conditional entropy $H_{c}$ for these stochastic systems increases monotonically to 0. To see what the temporal behavior of the Gibbs entropy is, consider first the one-dimensional Ornstein–Uhlenbeck process. If we start with a Gaussian density with mean $m_{0}$ and variance $\sigma_{0}^{2}$

$$f_{0}\left(v_{0}\right)=\frac{1}{\sqrt{2\pi \sigma_{0}^{2}}}\exp\left\{-\frac{{\left(v_{0}-m_{0}\right)}^{2}}{2\sigma_{0}^{2}}\right\},$$

then by Equations (17) and (15) we obtain

$$\bar{R}\left(t\right)=\sigma_{0}^{2}e^{-2\gamma t}+\int_{0}^{t}\sigma^{2}e^{-2\gamma s}ds=\sigma_{0}^{2}e^{-2\gamma t}+\frac{\sigma^{2}}{2\gamma }\left(1-e^{-2\gamma t}\right)$$

and the stationary density is given by

$$f_{*}\left(v\right)=\frac{1}{\sqrt{2\pi \sigma_{*}^{2}}}\exp\left\{-\frac{v^{2}}{2\sigma_{*}^{2}}\right\}\;\mathrm{with}\;\sigma_{*}^{2}=\frac{\sigma^{2}}{2\gamma }.$$

Thus, the Gibbs entropy is

$$H_{G}\left(f\left(\cdot ,t\right)\right)=\frac{1}{2}+\frac{1}{2}\ln\left(2\pi \right)+\frac{1}{2}\ln\left(\sigma_{*}^{2}+\left(\sigma_{0}^{2}-\sigma_{*}^{2}\right)e^{-2\gamma t}\right),$$

and it is either an increasing function of time when $\sigma_{0}^{2}>\sigma_{*}^{2}$ or a decreasing function of time when $\sigma_{0}^{2}<\sigma_{*}^{2}$, while the conditional entropy, given by

$$H_{c}\left(f\left(\cdot ,t\right)|f_{*}\right)=\frac{1}{2}\ln\left(1+\left(\frac{\sigma_{0}^{2}}{\sigma_{*}^{2}}-1\right)e^{-2\gamma t}\right)-\frac{1}{2}\left(\frac{\sigma_{0}^{2}}{\sigma_{*}^{2}}-1\right)e^{-2\gamma t}-\frac{m_{0}^{2}}{2\sigma_{*}^{2}}e^{-2\gamma t},$$

is a strictly increasing function of time for $\sigma_{0}^{2}\neq \sigma_{*}^{2}$ or $m_{0}\neq 0$.

**Figure 1:**
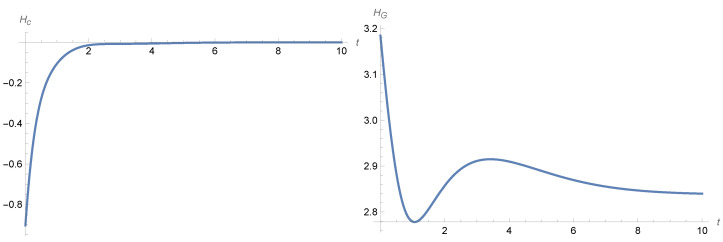
Entropy behaviour in the noisy harmonic oscillator (12). The **left**-hand panel shows the plot of the conditional entropy given by (24) and the **right** panel shows the plot of the Gibbs entropy given by (23). The parameters are γ=1, β=1/4, and $\sigma^{2}=1$ and the variance–covariance matrix *Q* is diagonal, with the initial variances of position and velocity being equal to 1 and 2 respectively, while the initial means are set to be equal to 0.

**Figure 2:**
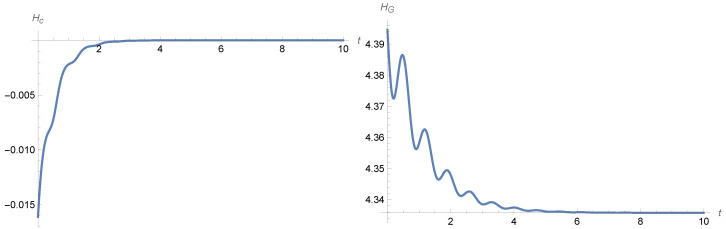
Entropy behaviour in the noisy harmonic oscillator (12). The **left**-hand panel shows the plot of the conditional entropy given by (24) and the **right** panel shows the plot of the Gibbs entropy given by (23). The parameters are γ=1, β=20, and $\sigma^{2}=40$ and the variance–covariance matrix *Q* is again diagonal, with the initial variances of position and velocity equal to 0.9 and 25 respectively, while the initial means are set equal to 0.

The temporal behavior of the Gibbs entropy can have more complicated patterns; see Figure 1 and Figure 2 and [1,2] in the case of the noisy harmonic oscillator.

## 4. Adding Delays in the Dynamics

Though the subject of the dynamics of differential delay equations (DDEs) is well developed and relatively mature, things are not quite so clear when one wishes to examine the role of delays in the temporal evolution of entropy. This is because in studying entropy dynamics, the *density* in a system plays a central role, and there are great difficulties in the study of density evolution under the action of delayed dynamics. Thus we investigate the temporal behavior of entropies for linear systems with additive perturbations leading to Gaussian processes with densities being determined through a vector-valued mean function and a matrix-valued variance–covariance function extending the one-dimensional linear differential equations with a single delay in [3]. We show that the temporal behavior of the conditional entropy depends on the initial condition and is not necessarily a monotone function of time.

The monograph [23] treats in detail the techniques that have been tried *unsuccessfully* to examine density evolution in delayed systems, while [24] examines certain aspects of this problem from the perspective of microphysical systems. Historically, important approaches have been made by a number of different groups ([24,25,26,27,28,29,30,31,32]), but it is unfortunately the situation that foundational issues are still unresolved and a satisfactory and unified theory allowing one to examine the evolution of densities in (infinite dimensional) delayed systems is yet to be developed. We [3,4] have employed various techniques to circumvent the problems outlined in [23] for the single delay case. At the end of this section, we show how this approach can be extended to functional differential equations with both discrete and distributed delays. The presentation is somewhat technical, but unavoidable, because of the difficulties encountered due to the infinite dimensional nature of systems with delayed dynamics.

### 4.1. Stochastic Functional Differential Equations

DDEs or more generally functional differential equations (FDEs) are equations in which the rate of change of a system at the present time depends not only on its current state but also on its state at one or more times in the past. The evolution of the system cannot be determined from a single initial value, because the equation explicitly refers to past states. Instead, one must specify a history function, which prescribes the state of the system over a whole time interval in the past—typically from time −τ up to 0, where τ is the maximum delay. This history function acts as the ‘initial condition’ for the differential equation: once it is given, the equation can be used to compute the solution forward in time, which motivates working on the space of paths that are continuous functions on the interval [−τ,0]. In this path-space setting, the solution map, denoted below as $S_{t}$, provides a clean way to describe a dynamical system showing how the initial function evolves forward in time. Because delayed dynamics are inherently non-Markovian at the level of the observed state, we need to work on this space of functions to recover the Markov property when adding noise. In Section 4.5 we recall the associated extended generator that captures the infinitesimal time evolution of observables on this enlarged space. Once this framework is in place, we can project back from paths to the observed state variable, recovering evolution equations for its probability density. We refer the reader to [33,34,35] for a thorough introduction to functional differential equations and their stochastic perturbations.

Suppose τ>0 is a given real number. For a continuous $x:\left[-\tau ,\infty \right)\to R^{d}$ we introduce the notation $x_{t}\in C$, where

$$x_{t}\left(s\right)=x\left(t+s\right)\;\mathrm{for}\;s\in \left[-\tau ,0\right],t\geq 0,$$

and $C:=C(\left[-\tau ,0\right],R^{d})$ is the space of continuous functions $\phi :\left[-\tau ,0\right]\to R^{d}$. Given a mapping $F:C\to R^{d}$ we consider the functional differential equation

(25) $$\left\{\begin{array}{c} x^{\prime }\left(t\right)=F\left(x_{t}\right),\;t\geq 0,\\ x_{0}=\phi . \end{array}\right.$$

We assume that *F* satisfies a global Lipschitz condition so that a unique solution $x:\left[-\tau ,\infty \right)\to R^{d}$ of (25) exists for each initial condition $x_{0}=\phi \in C$, and it depends continuously on (t,ϕ) and $x_{t}\in C$ for t>0. Then the solution map $S_{t}:C\to C$ given by

$$S_{t}\left(\phi \right)\left(s\right)=x_{t}\left(s\right)\;\mathrm{for}\;s\in \left[-\tau ,0\right],$$

is well defined for all t≥0 and gives rise to a semi-dynamical system ${\left\{S_{t}\right\}}_{t\in R^{+}}$ on the Banach space *C* equipped with the supremum norm [33]. In particular if

F(ϕ)=F(ϕ(0),ϕ(−τ)), ϕ∈C,

for some transformation $F:R^{d}\times R^{d}\to R^{d}$, then we recover the case of a single delay equation whose density evolution was studied in [3,4].

We next look at a noise-perturbed version of (25):

(26) $$\left\{\begin{array}{c} dx\left(t\right)=F\left(x_{t}\right)dt+\Sigma \left(x_{t}\right)dw\left(t\right),\;t\geq 0,\\ x_{0}=\phi , \end{array}\right.$$

where ${\left\{w\left(t\right)\right\}}_{t\geq 0}$ is again an *n*-dimensional standard Wiener process and the stochastic perturbation has an amplitude (diffusion coefficient) Σ, potentially continuously dependent on $x_{t}$, with values in the space $R^{n\times d}$ of n×d matrices. We assume that both *F* and Σ satisfy a global Lipschitz condition, so that the stochastic functional differential Equation (26) (see [35]) has a pathwise solution *x* and that ${\left\{x_{t}\right\}}_{t\geq 0}$ is a Markov process with values in the space *C*, where again

(27) $$x_{t}\left(s\right)=x\left(t+s\right),\;s\in \left[-\tau ,0\right],t\geq 0.$$

We end this section with a remark about how one can obtain a lower bound on the conditional entropy as in Section 3, but now for the densities defined by solutions of the stochastic functional differential equation considered in this section. If it happens that the solution $x\left(t\right)=x_{t}\left(0\right)$ of (26) with each deterministic initial condition ϕ∈C has a probability density function, say p(x,t|ϕ), then the function

$$f\left(x,t\right)=\int_{C}p\left(x,t|\phi \right)\mu_{0}\left(d\phi \right),\;x\in R^{d},t\geq 0,$$

defines the density of $x\left(t\right)=x_{t}\left(0\right)$ when the initial condition $x_{0}$ has the distribution $\mu_{0}$, which is now a distribution on the function space *C*. Using Jensen’s inequality, we obtain [3]

$$H_{c}\left(f\left(\cdot ,t\right)|f_{*}\right)\geq \int_{C}H_{c}\left(p\left(\cdot ,t|\phi \right)|f_{*}\right)\mu_{0}\left(d\phi \right).$$

Consequently, if $H_{c}\left(p\left(\cdot ,t|\phi \right)|f_{*}\right)$ converges to zero as t→∞ for all ϕ∈C then we conclude that

$$\lim_{t\to \infty }H_{c}\left(f\left(\cdot ,t\right)|f_{*}\right)=0.$$

### 4.2. A Linear Functional Differential Equation

A particular example of a linear functional differential equation is

$$x^{\prime }\left(t\right)=Ax\left(t\right)+Bx\left(t-\tau \right)+\int_{-\tau }^{0}A\left(\theta \right)x\left(t+\theta \right)d\theta ,$$

where *A* and *B* are d×d matrices and $A\left(\cdot \right):\left[-\tau ,0\right]\to R^{d\times d}$ is a continuous matrix-valued function. Once again, to proceed, we require some technical developments specific to linear delayed dynamics.

Suppose that $F:C\to R^{d}$ is a continuous linear mapping. By the Riesz representation theorem, we can write

$$F\left(\phi \right)=A\phi \left(0\right)+\int_{-\tau }^{0}d\eta \left(\theta \right)\phi \left(\theta \right),$$

where $\eta ={\left[\eta_{ij}\right]}_{i,j=1,\ldots ,d}$ is a matrix-valued function of bounded variation and the integral is the Lebesgue–Stieltjes integral. Note that F(ϕ)−Aϕ(0) is a *d*-dimensional vector with coordinates

$$\sum_{j=1}^{d}\int_{-\tau }^{0}\phi_{j}\left(\theta \right)d\eta_{ij}\left(\theta \right),\;i=1,\ldots ,d.$$

Let $S_{t}:C\to C$ be the solution map of the functional linear equation

(28) $$\left\{\begin{array}{c} x^{\prime }\left(t\right)=Ax\left(t\right)+\int_{-\tau }^{0}d\eta \left(\theta \right)x\left(t+\theta \right),\;t\geq 0,\\ x_{0}=\phi . \end{array}\right.$$

In particular, we have

$$x\left(t\right)=S_{t}\phi \left(0\right),\;t\geq 0.$$

The stability of the trivial solution x(t)≡0 is determined by the roots of the characteristic (eigenvalue) equation

(29) $$\det h\left(\lambda \right)=0,\;h\left(\lambda \right)=\lambda I-A-\int_{-\tau }^{0}e^{\theta \lambda }d\eta \left(\theta \right),$$

where $h:C\to C^{d\times d}$. If all solutions of (29) have negative real parts, then all solutions of (28) tend to 0 as t→∞.

The fundamental solution X(t) of Equation (28) is the d×d matrix function satisfying

(30) $$X^{\prime }\left(t\right)=AX\left(t\right)+\int_{-\tau }^{0}d\eta \left(\theta \right)X\left(t+\theta \right)$$

with

(31) X(s)=0 for s∈[−τ,0) and X(0)=I,

where *I* is the identity matrix. Let

(32) α0=sup{Re(λ):deth(λ)=0}.

As in the scalar case the fundamental matrix solution *X* has the following bound [33]: for each $\alpha >\alpha_{0}$ there exists a constant C>0 such that

(33) $$\left|X\left(t\right)\right|\leq Ce^{\alpha t}\;\mathrm{for}\;\mathrm{all}\;t>0,$$

where |·| is a norm of the d×d matrix.

Let Σ be a d×n matrix and *w* be an *n*-dimensional standard Wiener process. Given an initial continuous function $\phi :\left[-\tau ,0\right]\to R^{d}$ we can write the solution of

(34) $$\left\{\begin{array}{c} dx\left(t\right)=(Ax\left(t\right)+\int_{-\tau }^{0}d\eta \left(\theta \right)x\left(t+\theta \right))dt+\Sigma dw\left(t\right),\;t>0,\\ x_{0}=\phi , \end{array}\right.$$

as

$$x\left(t\right)=S_{t}\phi \left(0\right)+\int_{0}^{t}X\left(t-r\right)\Sigma dw\left(r\right),$$

where $S_{t}$ is the solution map and X(t) is the fundamental matrix solution of the deterministic part (28). This corresponds to (14) in the case of τ=0. The process

$$\zeta \left(t\right)=\int_{0}^{t}X\left(t-r\right)\Sigma dw\left(r\right),\;t\geq 0,$$

is a Gaussian process with values in $R^{d}$ that have zero mean vector and the matrix-valued covariance function of the form

$$E\left(\zeta \left(t\right)\zeta {\left(t+s\right)}^{T}\right)=\int_{0}^{t}X\left(t-r\right)\Sigma \Sigma^{T}X{\left(t+s-r\right)}^{T}dr,\;t,s\geq 0,$$

by Itô’s isometry. The variance of ζ(t) is thus given by

(35) $$R\left(t\right):=\mathrm{Var}\left(\zeta \left(t\right)\right)=E\left(\zeta \left(t\right)\zeta {\left(t\right)}^{T}\right)=\int_{0}^{t}X\left(r\right)\Sigma \Sigma^{T}X{\left(r\right)}^{T}dr,\;t\geq 0.$$

Note that R(t) is as in (15) when τ=0. Although the matrix R(t) in (35) is symmetric and non-negative definite, it does not need be positive definite. Observe that ζ defines for each t≥0 the segment $\zeta_{t}$ by

$$\zeta_{t}\left(s\right)=\left\{\begin{array}{cc} \zeta (t+s), & \mathrm{if}\;s+t\geq 0,s\in [-\tau ,0],\\ 0, & \mathrm{if}\;s+t<0,s\in [-\tau ,0]. \end{array}\right.$$

Consequently, the process $x_{t}$ in (27) being defined by the solution of (34) has the following representation:

(36) $$x_{t}=S_{t}\phi +\zeta_{t},\;x_{0}=\phi .$$

Let $\mu_{t}$ be the distribution of $x_{t}$ as in (36). Now suppose that $x_{0}=\phi$ is a Gaussian process. Then $x_{t}$ is a Gaussian process and x(t) is an $R^{d}$-valued Gaussian random vector. To characterize its distribution μ(t) it is enough to find its mean vector and the variance–covariance matrix. We have

$$E\left(x\left(t\right)\right)=S_{t}m_{0}\left(0\right),$$

where $S_{t}m_{0}$ is the solution of (28) with the initial condition being the mean function

$$m_{0}\left(s\right)=E\left(\phi \left(s\right)\right),\;s\in \left[-\tau ,0\right],$$

and the variance of x(t) is given by the equation

(37) $$\bar{R}\left(t\right):=\mathrm{Var}\left(x\left(t\right)\right)=\mathrm{Var}\left(S_{t}\phi \left(0\right)\right)+R\left(t\right),\;t>0.$$

In particular, if $x_{0}=\phi$ is deterministic, so that the function ϕ is fixed, then $x_{t}$ is also a Gaussian process and $\mathrm{Var}(S_{t}\phi \left(0\right))=0$. If, on the other hand, $S_{t}\phi$ is an $R^{d}$-valued Gaussian process with mean vector $S_{t}m_{0}$ then we can assume that $m_{0}\equiv 0$.

#### 4.2.1. Existence of a Stationary Density

Suppose that $\alpha_{0}<0$, where $\alpha_{0}$ is defined in (32). Then $S_{t}\phi \left(0\right)\to 0$ as t→∞. It follows from (33) that for some α<0 and C>0

$$|\int_{t}^{\infty }X\left(r\right)\Sigma \Sigma^{T}X{\left(r\right)}^{T}dr|\leq C\left|\Sigma \Sigma^{T}\right|\int_{t}^{\infty }e^{2\alpha r}dr\to 0\;\mathrm{as}\;t\to \infty ,$$

implying that the following limit exists and is finite:

(38) $$\lim_{t\to \infty }R\left(t\right)=\int_{0}^{\infty }X\left(r\right)\Sigma \Sigma^{T}X{\left(r\right)}^{T}dr:=R_{*}.$$

If R(t) is non-singular (equivalently, R(t) is positive definite), then the density *p* of the distribution μ(t) of x(t) is given by the Gaussian density

(39) $$p\left(x,t|\phi \right)=\frac{1}{{\left(2\pi \right)}^{d/2}{\left(\det R\left(t\right)\right)}^{1/2}}e^{-\frac{1}{2}{\left(x-S_{t}\phi \left(0\right)\right)}^{T}R{\left(t\right)}^{-1}\left(x-S_{t}\phi \left(0\right)\right)},\;x\in R^{d},$$

where $R{\left(t\right)}^{-1}$ is the inverse matrix of the covariance matrix R(t). If R(t) is positive definite, we see that its limit $R_{*}$ is also positive definite and thus invertible. Then we have

(40) $$\lim_{t\to \infty }p\left(x,t|\phi \right)=\frac{1}{{\left(2\pi \right)}^{d/2}{\left(\det R_{*}\right)}^{1/2}}e^{-\frac{1}{2}x^{T}R_{*}^{-1}x}=f_{*}\left(x\right),$$

and $f_{*}$ is as in (20) with $R_{*}$ now given by (38). Equation (34) has a stationary solution of the form [35] (Corollary 4.2.1)

(41) $$Z\left(s\right)=\int_{-\infty }^{s}X\left(s-r\right)\Sigma dw\left(r\right),\;s\in R$$

and the distribution of Z(s) is Gaussian with density $f_{*}$ as in (40).

#### 4.2.2. The Gibbs and Conditional Entropies

In this section, we assume that the matrix R(t) is positive definite for all sufficiently large *t*. Let the initial condition ϕ be a Gaussian process and let f(·,t) be the Gaussian density of x(t) with variance $\bar{R}\left(t\right)$ as in (37) and the mean vector $S_{t}m_{0}$ with $m_{0}$ being the mean vector of the initial condition ϕ. By (21) we have

(42) $$H_{G}\left(f\left(\cdot ,t\right)\right)=\frac{1}{2}\left\{d+\left[\ln{\left(2\pi \right)}^{d}\det\bar{R}\left(t\right))\right]\right\},$$

and thus

$$\frac{dH_{G}\left(f\left(\cdot ,t\right)\right)}{dt}=\frac{1}{2\det\bar{R}\left(t\right)}\frac{d\det\bar{R}\left(t\right)}{dt}.$$

By Jacobi’s formula we have

$$\frac{d\ln\det\bar{R}\left(t\right)}{dt}=\mathrm{tr}\left(\bar{R}{\left(t\right)}^{-1}\frac{d\bar{R}\left(t\right)}{dt}\right),$$

implying that

$$\frac{dH_{G}\left(f\left(\cdot ,t\right)\right)}{dt}=\frac{1}{2}\mathrm{tr}\left(\bar{R}{\left(t\right)}^{-1}\frac{d\bar{R}\left(t\right)}{dt}\right).$$

If we take $f_{m_{1},Q_{1}}$ as in (39) and $f_{m_{2},Q_{2}}$ as in (40), then $Q_{1}=\bar{R}\left(t\right)$, $m_{1}=S_{t}m_{0}\left(0\right)$, and $Q_{2}=R_{*}$, $m_{2}=0$. Hence, by (22), we have

(43) $$\begin{array}{c} H_{c}\left(f\left(\cdot ,t\right)|f_{*}\right)=\frac{1}{2}\ln\det\left(R_{*}^{-1}\bar{R}\left(t\right)\right)+\frac{1}{2}\mathrm{tr}\left[I-R_{*}^{-1}\bar{R}\left(t\right)\right]\\ -\frac{1}{2}\mathrm{tr}\left[R_{*}^{-1}S_{t}m_{0}\left(0\right){\left(S_{t}m_{0}\left(0\right)\right)}^{T}\right]\; \end{array}$$

and so the temporal derivative of the conditional entropy satisfies

(44) $$\frac{dH_{c}\left(f\left(\cdot ,t\right)|f_{*}\right)}{dt}=\frac{1}{2}\mathrm{tr}\left(\bar{R}{\left(t\right)}^{-1}\frac{d\bar{R}\left(t\right)}{dt}-R_{*}^{-1}\frac{d\bar{R}\left(t\right)}{dt}-R_{*}^{-1}\frac{dS_{t}m_{0}\left(0\right){\left(S_{t}m_{0}\left(0\right)\right)}^{T}}{dt}\right).$$

If we take as the initial condition $x_{0}=\phi$ with $\phi \left(s\right)=Z\left(s\right)+m_{0}\left(s\right)$ for s∈[−θ,0], where *Z* is the Gaussian process, being the stationary solution of (34), and $m_{0}$ is a non-zero initial mean function, then $\bar{R}\left(t\right)=R_{*}$ and we obtain from (43) that

(45) $$H_{c}\left(f\left(\cdot ,t\right)|f_{*}\right)=-\frac{1}{2}\mathrm{tr}\left[R_{*}^{-1}S_{t}m_{0}\left(0\right){\left(S_{t}m_{0}\left(0\right)\right)}^{T}\right].$$

However, we cannot conclude that the conditional entropy $H_{c}$, given by the function $-\frac{1}{2}\mathrm{tr}\left[R_{*}^{-1}S_{t}m_{0}\left(0\right){\left(S_{t}m_{0}\left(0\right)\right)}^{T}\right]$, is strictly increasing although it converges to zero. See Section 4.3 for examples.

If, on the other hand, we take $m_{0}=\phi$ with ϕ fixed, so that the initial condition is deterministic, then $\bar{R}\left(t\right)=R\left(t\right)$. Hence, we recover $H_{G}\left(p\left(\cdot ,t|\phi \right)\right)$ from Equation (42) and $H_{c}\left(p\left(\cdot ,t|\phi \right)|f_{*}\right)$ from (43). Since the matrix $\frac{dR(t)}{dt}$ is non-negative definite, we see that the matrix $R{\left(t\right)}^{-1}\frac{dR(t)}{dt}$ is also nonnegative definite. Consequently, its trace is nonnegative, implying that

(46) $$\frac{dH_{G}\left(p\left(\cdot ,t|\phi \right)\right)}{dt}\geq 0$$

so *the Gibbs entropy of the density p is monotone increasing and thus the strong form of the second law holds*. Since

$$R{\left(t\right)}^{-1}\frac{dR(t)}{dt}-R_{*}^{-1}\frac{dR(t)}{dt}=R_{*}^{-1}\left(R_{*}-R\left(t\right)\right)R{\left(t\right)}^{-1}\frac{dR(t)}{dt}$$

and the matrix $R_{*}-R\left(t\right)$, given by

$$R_{*}-R\left(t\right)=\int_{t}^{\infty }X\left(r\right)\Sigma \Sigma^{T}X{\left(r\right)}^{T}dr,$$

is non-negative definite, we conclude that

$$\mathrm{tr}\left(R{\left(t\right)}^{-1}\frac{dR(t)}{dt}-R_{*}^{-1}\frac{dR(t)}{dt}\right)\geq 0.$$

However, as in the one-dimensional case we cannot claim that

$$-\mathrm{tr}\left(R_{*}^{-1}\frac{dS_{t}\phi \left(0\right){\left(S_{t}\phi \left(0\right)\right)}^{T}}{dt}\right)\geq 0$$

and once again *the presence of the delay has opened the possibility of a non-monotone approach of the conditional entropy to zero and, thus, a shift to the weak form of the second law*.

Finally, for the density f(x,t) of $x_{t}\left(0\right)$ with initial condition $x_{0}$ chosen according to a distribution $\mu_{0}$ we obtain

$$H_{c}\left(f\left(\cdot ,t\right)|f_{*}\right)\geq \frac{1}{2}\ln\;\frac{\det R(t)}{\det R_{*}}+\frac{1}{2}\mathrm{tr}\left[I-R_{*}^{-1}R\left(t\right)\right]-\frac{1}{2}\int_{C}\mathrm{tr}\left[R_{*}^{-1}S_{t}\phi \left(0\right){\left(S_{t}\phi \left(0\right)\right)}^{T}\right]\mu_{0}\left(d\phi \right).$$

Since $R\left(t\right)\to R_{*}$ and $S_{t}\phi \left(0\right)\to 0$ as t→∞, we conclude that

(47) $$\lim_{t\to \infty }H_{c}\left(f\left(\cdot ,t\right)|f_{*}\right)=0.$$

#### 4.2.3. The Covariance of the Stationary Solution

To find $R_{*}$ let us look at the matrix-valued covariance of the process {Z(s):s∈R}:

$$K\left(s\right)=E(Z\left(t\right)Z{\left(s+t\right)}^{T}),\;s\in R.$$

The covariance function is given by

$$K\left(s\right)=\int_{-\infty }^{\min\{t,s+t\}}X\left(t-u\right)\Sigma \Sigma^{T}X{\left(s+t-u\right)}^{T}du$$

by the Itô isometry. Changing the variables in the integral leads to

$$K\left(s\right)=\int_{0}^{\infty }X\left(r\right)\Sigma \Sigma^{T}X{\left(r+s\right)}^{T}dr,\;s\geq 0,$$

and

$$K\left(s\right)=\int_{0}^{\infty }X\left(r-s\right)\Sigma \Sigma^{T}X{\left(r\right)}^{T}dr,\;s<0.$$

Hence $K\left(s\right)=K{\left(-s\right)}^{T}$ for s<0. Note that

$$R_{*}=K\left(0\right)=K{\left(0\right)}^{T}.$$

Let us now derive a differential equation for the matrix K(t) for t>0. We have

$$K^{\prime }\left(t\right)=\int_{0}^{\infty }X\left(r\right)\Sigma \Sigma^{T}X^{\prime }{\left(r+t\right)}^{T}dr.$$

It follows from (30) that

$$X^{\prime }{\left(r+t\right)}^{T}=X{\left(r+t\right)}^{T}A^{T}+\int_{-\tau }^{0}X{\left(r+t+\theta \right)}^{T}d\eta^{T}\left(\theta \right),\;r,t>0,$$

implying that *K* satisfies the equation

(48) $$K^{\prime }\left(t\right)=K\left(t\right)A^{T}+\int_{-\tau }^{0}K\left(t+\theta \right)d\eta^{T}\left(\theta \right),\;t>0.$$

Moreover, we have

$$\frac{d}{dr}\left(X\left(r\right)\Sigma \Sigma^{T}X{\left(r\right)}^{T}\right)=X^{\prime }\left(r\right)\Sigma \Sigma^{T}X{\left(r\right)}^{T}+X\left(r\right)\Sigma \Sigma^{T}X^{\prime }{\left(r\right)}^{T}$$

and

$$\int_{0}^{\infty }X\left(r\right)\Sigma \Sigma^{T}X^{\prime }{\left(r\right)}^{T}dr=K^{\prime }\left(0\right),\;\mathrm{where}\;K^{\prime }\left(0\right)=\lim_{s↓0}K^{\prime }\left(s\right).$$

Hence, we obtain

(49) $$K^{\prime }{\left(0\right)}^{T}+K^{\prime }\left(0\right)=-\Sigma \Sigma^{T}\;\mathrm{with}\;K^{\prime }\left(0\right)=K\left(0\right)A^{T}+\int_{-\tau }^{0}K\left(\theta \right)d\eta^{T}\left(\theta \right).$$

Consequently, if we want to find $R_{*}=K\left(0\right)$, then we need to solve Equation (48) subject to condition (49).

In general, it is impossible to find a solution of (48), though in the one-dimensional case with a single discrete delay one was derived in [36]; see also [28]. Here we obtain a second-order differential equation for the covariance *K* of the stationary solution of equation

(50) dx(t)=[Ax(t)+Bx(−τ)]dt+Σdw(t),

where *B* is a d×d matrix. It follows from (48) that the covariance K(t) of the stationary solution satisfies

$$K^{\prime }\left(t\right)=K\left(t\right)A^{T}+K\left(t-\tau \right)B^{T},\;t>0,$$

and $K\left(s\right)=K{\left(-s\right)}^{T}$ for s≤0. For t∈(0,τ) we have $K\left(t-\tau \right)=K{\left(\tau -t\right)}^{T}$. Thus, we obtain

(51) $$K^{\prime }\left(t\right)=K\left(t\right)A^{T}+K{\left(\tau -t\right)}^{T}B^{T},\;t\in \left(0,\tau \right).$$

Next, differentiating the function $U\left(t\right)=K{\left(\tau -t\right)}^{T}$ we get

$$U^{\prime }\left(t\right)=\frac{d}{dt}\left[K{\left(\tau -t\right)}^{T}\right]=-AK{\left(\tau -t\right)}^{T}-BK\left(t\right)$$

implying that

$$K^{\prime \prime }\left(t\right)=K^{\prime }\left(t\right)A^{T}+U^{\prime }\left(t\right)B^{T}=K^{\prime }\left(t\right)A^{T}-AK{\left(\tau -t\right)}^{T}B^{T}-BK\left(t\right)B^{T}.$$

From Equation (51) we obtain

$$K{\left(\tau -t\right)}^{T}B^{T}=K^{\prime }\left(t\right)-K\left(t\right)A^{T}.$$

Consequently, the covariance satisfies for t∈(0,τ) the second-order matrix differential equation

(52) $$K^{\prime \prime }\left(t\right)=K^{\prime }\left(t\right)A^{T}-AK^{\prime }\left(t\right)+AK\left(t\right)A^{T}-BK\left(t\right)B^{T}$$

with

(53) $$K^{\prime }\left(0\right)+K^{\prime }{\left(0\right)}^{T}=-\Sigma \Sigma^{T},\;K^{\prime }\left(0\right)=K\left(0\right)A^{T}+K{\left(\tau \right)}^{T}B^{T}.$$

Let us summarize symbols used in Section 4.2:

- *F*—a general linear continuous mapping on $C=C\left([-\tau ,0\right],R^{d})$, *A*, *B* – d×d matrices;
- $S_{t}$—the solution map of (28); $S_{t}\phi \left(0\right)$ is the solution of (28);
- X(t)—the fundamental matrix solution (30) and (31);
- x(t)—the solution of (34); its variance is $\bar{R}\left(t\right)$;
- ζ(t)—a Gaussian vector with zero mean and the variance equal to R(t) as in (35);
- $R_{*}$—the limit of the variance R(t) as defined in (38);
- Z(s),s∈R—the Gaussian process as in (41) that is the stationary solution of (34);
- K(s)—the matrix-valued covariance function of the stationary process *Z*; $K\left(0\right)=R_{*}$.

### 4.3. Examples

#### 4.3.1. A Linear Equation with Single Delay in One Dimension

Consider the single delay Equation (50) in the case of d=1, so that A=a and B=b are real constants

dx(t)=(ax(t)+bx(t−τ))dt+σdw(t).

**Figure 3:**
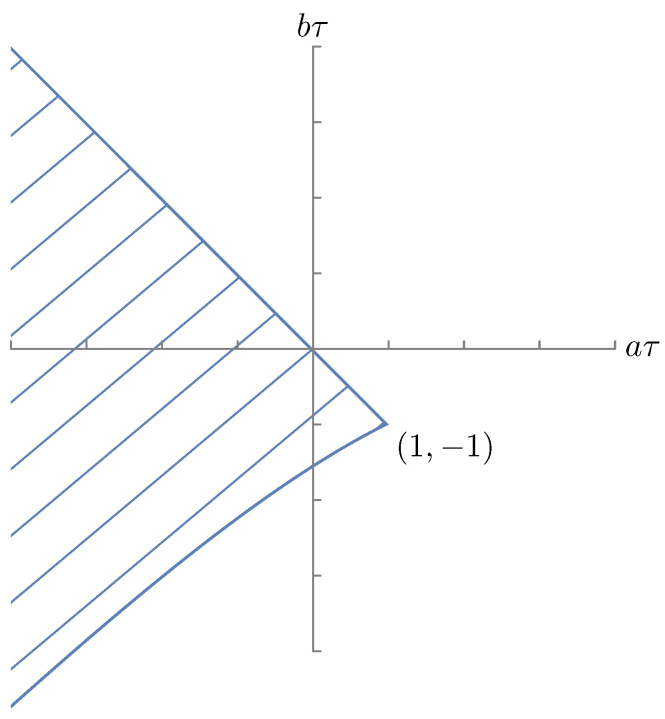
A graphical representation of the stability region for the linear equation with single delay as determined by [37]. Modified from [38].

The entirety of the (aτ,bτ) plane inside the hatched wedge-shaped area in Figure 3 corresponds to parameter values (a,b,τ) such that this equation has a stationary solution and a stationary density.

Equation (52) for the variance K(t) of the stationary solution *Z* reduces to

$$K^{\prime \prime }\left(t\right)=\left(a^{2}-b^{2}\right)K\left(t\right),$$

with initial and boundary conditions

$$K^{\prime }\left(0\right)=-\frac{\sigma^{2}}{2},\;K^{\prime }\left(0\right)=aK\left(0\right)+bK\left(\tau \right).$$

It follows from [36] that if $a^{2}\neq b^{2}$ then the variance $K\left(0\right)=V_{*}$ is given by

$$V_{*}=\frac{\sigma^{2}}{2}\frac{b\sinh\left(\sqrt{a^{2}-b^{2}}\tau \right)-\sqrt{a^{2}-b^{2}}}{\sqrt{a^{2}-b^{2}}\left(b\cosh\left(\sqrt{a^{2}-b^{2}}\tau \right)+a\right)}.$$

**Figure 4:**
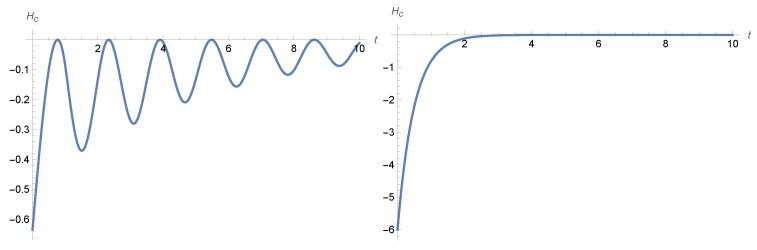
The **left**-hand panel shows the temporal behaviour of the conditional entropy given by (54) for an initial condition $m_{0}\left(s\right)=s+1$ with s∈[−1,0] and parameters a=−1, b=−2, τ=1, and σ=1, while the **right**-hand panel shows the plot of the increasing conditional entropy given by (54) for τ=0, b=0, and initial condition $m_{0}=1$.

Note that if τ=0, b=0 and a=−γ we recover the variance $\sigma_{*}^{2}$ of the stationary Ornstein–Uhlenbeck process from Section 3.2. The conditional entropy as given in (45) reduces to

(54) $$H_{c}\left(f\left(\cdot ,t\right)|f_{*}\right)=-\frac{1}{2}\frac{m^{2}\left(t\right)}{\sigma_{*}^{2}},$$

where $m\left(t\right)=S_{t}m_{0}\left(0\right)$ is the solution of the equation $x^{\prime }\left(t\right)=ax\left(t\right)+bx\left(t-\tau \right)$ with the initial condition function $m_{0}\left(s\right)$, s∈[−τ,0]; see Figure 4.

#### 4.3.2. A Second-Order Equation with One Delay

We now consider a particle of mass *m*, located at a position x(t) and moving with velocity v(t) and subject to a delayed restoring force [−βx(t−τ)] as well as a delayed frictional force −γv(t−τ) and a fluctuating force σξ(t), where β>0 and γ>0, while σ≥0 and ξ(t) is the white noise. Thus, the dynamics of this noisy, delayed harmonic oscillator are given by

(55) $$\left\{\begin{array}{c} \frac{d}{dt}x\left(t\right)=v\left(t\right),\\ m\frac{d}{dt}v\left(t\right)=-\gamma v\left(t-\tau \right)-\beta x\left(t-\tau \right)+\sigma \xi \left(t\right). \end{array}\right.$$

Note that the system (55) with m=1 in the absence of noise (σ=0) is equivalent to the second-order linear delayed equation

(56) $$\frac{d^{2}}{dt^{2}}x\left(t\right)=-\gamma \frac{d}{dt}x\left(t-\tau \right)-\beta x\left(t-\tau \right).$$

This equation is a special case of the general second-order delay differential equation

(57) $$\frac{d^{2}}{dt^{2}}x\left(t\right)+a_{1}\frac{d}{dt}x\left(t\right)+a_{0}x\left(t\right)=b_{0}x\left(t-\tau \right)+b_{1}\frac{d}{dt}x\left(t-\tau \right),$$

where $a_{1},a_{0},b_{1},b_{0}$ are real constants. The stability of the trivial solution x(t)≡0 of the deterministic Equation (57) is determined by the roots of the characteristic (eigenvalue) equation

(58) $$\lambda^{2}+a_{1}\lambda +a_{0}-b_{1}\lambda e^{-\lambda \tau }-b_{0}e^{-\lambda \tau }=0,$$

obtained with the ansatz $x\left(t\right)∼e^{\lambda t}$. One can infer conditions for asymptotic stability from the literature [39,40,41,42]. In particular, if $b_{0}\geq a_{0}$ then the trivial solution is unstable. In what follows we thus assume that $a_{0}>b_{0}$ and that all roots of (58) have negative real parts, i.e.,

a0>b0 and α0:=sup{Re(λ):λ satisfies (58)}<0.

**Figure 5:**
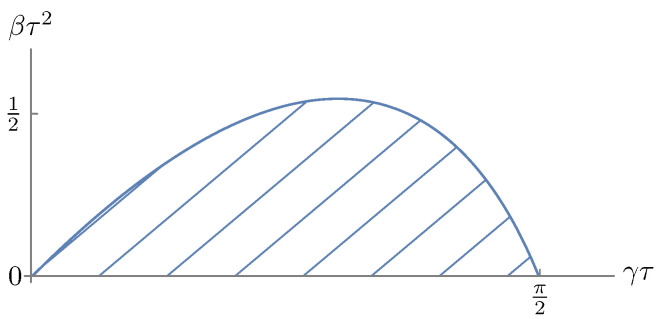
A graphical representation of the stability region of the steady state x≡0 of (56). The entirety of the $\left(\gamma \tau ,\beta \tau^{2}\right)$ plane in the hatched banana-shaped area corresponds to parameter values (β,γ,τ) such that x=0 is stable. Modified from Kolmanovskii and Myshkis [34] (Chapter 4).

Note that if we take τ=0 in (58), then we have $\alpha_{0}<0$ iff $a_{0}>b_{0}$ and $a_{1}>b_{1}$. See Section 4.4.2 for necessary and sufficient conditions for $\alpha_{0}<0$ when τ>0. The stability region of parameters for Equation (56) is presented in Figure 5.

Now consider Equation (57) with noise

$$\frac{d^{2}}{dt^{2}}x\left(t\right)+a_{1}\frac{d}{dt}x\left(t\right)+a_{0}x\left(t\right)=b_{0}x\left(t-\tau \right)+b_{1}\frac{d}{dt}x\left(t-\tau \right)+\sigma \xi \left(t\right),$$

and see [29,31,32] for particular examples. Introducing the variables

$$x\left(t\right)=\left(\begin{array}{c} x(t)\\ v(t) \end{array}\right)\;\mathrm{with}\;v\left(t\right)=x^{\prime }\left(t\right),$$

then we obtain

(59) dx(t)=[Ax(t)+Bx(t−τ)]dt+Σdw(t),

where w(t) is a standard one-dimensional Wiener process and

$$A=\left(\begin{array}{cc} 0 & 1\\ -a_{0} & -a_{1} \end{array}\right),\;B=\left(\begin{array}{cc} 0 & 0\\ b_{0} & b_{1} \end{array}\right),\;\Sigma =\left(\begin{array}{c} 0\\ \sigma \end{array}\right).$$

We can define a stationary solution {Z(t):t∈R} for (59) as in the previous section. Let $K\left(t\right)={\left(k_{ij}\left(t\right)\right)}_{i,j=1,2}$ be its covariance matrix t≥0.

First, we show that

(60) $$k_{12}\left(t\right)+k_{21}\left(t\right)=0\;\mathrm{for}\;\mathrm{all}\;t\geq 0.$$

Recall that

(61) $$K\left(t\right)=\int_{0}^{\infty }X\left(r\right)\Sigma \Sigma^{T}X{\left(r+t\right)}^{T}dr.$$

Since the fundamental solution X(t) is a 2×2 matrix, which we represent as $X\left(t\right)={\left(x_{ij}\left(t\right)\right)}_{i,j=1,2}$, and furthermore, it satisfies

$$X^{\prime }\left(t\right)=AX\left(t\right)+BX\left(t-\tau \right),\;t>0,X\left(0\right)=I,\;X\left(s\right)=0\;\mathrm{for}\;s<0,$$

we see that

$$x_{12}^{\prime }\left(t\right)=x_{22}\left(t\right)\;\mathrm{for}\;t>0,\;x_{12}\left(s\right)=0\;\mathrm{for}\;s\leq 0.$$

Thus, we obtain

$$X\left(r\right)\Sigma =\sigma \left(\begin{array}{c} x_{12}\left(r\right)\\ x_{12}^{\prime }\left(r\right) \end{array}\right)$$

and

$$X\left(r\right)\Sigma \Sigma^{T}X{\left(r+t\right)}^{T}=\sigma^{2}\left(\begin{array}{cc} x_{12}\left(r\right)x_{12}\left(r+t\right) & x_{12}\left(r\right)x_{12}^{\prime }\left(r+t\right)\\ x_{12}^{\prime }\left(r\right)x_{12}\left(r+t\right) & x_{12}^{\prime }\left(r\right)x_{12}^{\prime }\left(r+t\right) \end{array}\right).$$

We have

$$k_{12}\left(t\right)=\int_{0}^{\infty }\sigma^{2}x_{12}\left(r\right)x_{12}^{\prime }\left(r+t\right)dr.$$

Since $x_{12}\left(r\right)\to 0$ as r→∞ and $x_{12}\left(0\right)=0$, integrating by parts leads to

$$k_{12}\left(t\right)=-\int_{0}^{\infty }x_{12}^{\prime }\left(r\right)x_{12}\left(r+t\right)dr=-k_{21}\left(t\right).$$

Next it follows from (60) that the matrix $R_{*}=K\left(0\right)$ is diagonal, since $K{\left(0\right)}^{T}=K\left(0\right)$. Thus the stationary density $f_{*}$ as given by (40) will be of the form

$$f_{*}\left(x,v\right)=\frac{1}{2\pi \sqrt{\sigma_{x}^{2}\sigma_{v}^{2}}}e^{-\left(\frac{x^{2}}{2\sigma_{x}^{2}}+\frac{v^{2}}{2\sigma_{v}^{2}}\right)},\;x,v\in R,$$

where the variances

(62) $$\sigma_{x}^{2}=k_{11}\left(0\right)\;\mathrm{and}\;\sigma_{v}^{2}=k_{22}\left(0\right)$$

and $k_{11}\left(0\right)$, $k_{22}\left(0\right)$ are the diagonal elements of the matrix K(0) as given by (85).

The Gibbs and conditional entropies behave as described in Section 4.2. Although the conditional entropy always converges to zero, it might not be an increasing function of time, as the following example shows.

**Figure 6:**
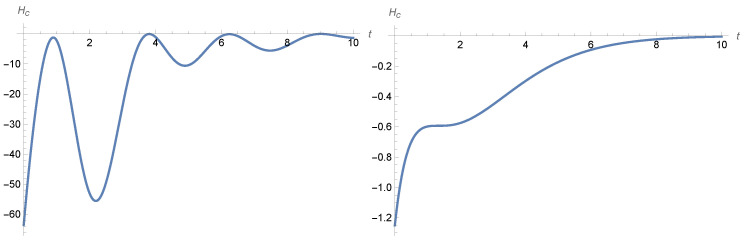
The **left**-hand panel shows a plot of the conditional entropy given by (63) for initial condition $m_{0x}\left(s\right)=s+1$ for s∈[−1,0] and parameters $a_{0}=a_{1}=0$, $b_{0}=-0.25$, $b_{1}=-1$, τ=1, σ=1, while the **right**-hand panel shows the plot of the conditional entropy, given by (63), with τ=0 for initial condition $m_{x}\left(0\right)=1$, $m_{v}\left(0\right)=1$.

**Figure 7:**
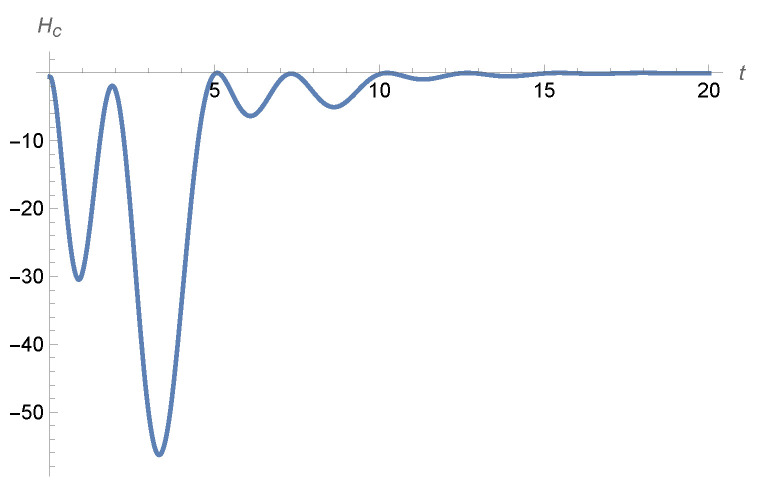
This is a plot of the conditional entropy as given by (63) for initial condition $m_{0x}\left(s\right)=s^{2}+1$ for s∈[−1,0] and parameters $a_{0}=a_{1}=0$, $b_{0}=-0.25$, $b_{1}=-1$, τ=1, σ=1.

We consider the initial condition $x_{0}$ to be $x_{0}\left(s\right)=Z\left(s\right)+m_{0}\left(s\right)$, s∈[−τ,0], where *Z* is the stationary Gaussian process and $m_{0}$ is a non-zero mean vector function with $m_{0}\left(s\right)={\left(m_{0x}\left(s\right),m_{0x}^{\prime }\left(s\right)\right)}^{T}$, s∈[−τ,0]. Then the conditional entropy is by (45), equal to

(63) $$H_{c}\left(f\left(\cdot ,t\right)|f_{*}\right)=-\frac{1}{2}\left(\frac{m_{x}^{2}\left(t\right)}{\sigma_{x}^{2}}+\frac{m_{v}^{2}\left(t\right)}{\sigma_{v}^{2}}\right),$$

where $m_{x}\left(t\right)$ is the solution of (57) with initial condition $m_{0x}$, while $m_{v}\left(t\right)=m_{x}^{\prime }\left(t\right)$. Particular examples are given in Figure 6 and Figure 7, where we have $a_{0}=0$ and $a_{1}=0$ together with $b_{0}=-\beta$ and $b_{1}=-\gamma$ with the values of (β,γ,τ) in the hatched area of Figure 5.

### 4.4. Determination of the Variances in *(62)*

This section is devoted to a derivation of the variances $k_{11}\left(0\right)$ and $k_{22}\left(0\right)$ as functions of parameters $a_{1},a_{0},b_{1},b_{0}$ and the delay τ for the stationary solution of (59). Let $K\left(t\right)={\left(k_{ij}\left(t\right)\right)}_{i,j=1,2}$ be its covariance matrix function (61). To find the equation for $k_{ij}\left(t\right)$ for t∈(0,τ) we infer from (51) and (60) that

$$\begin{array}{c} \left(\begin{array}{cc} k_{11}^{\prime }\left(t\right) & -k_{21}^{\prime }\left(t\right)\\ k_{21}^{\prime }\left(t\right) & k_{22}^{\prime }\left(t\right) \end{array}\right)=\left(\begin{array}{cc} k_{11}\left(t\right) & -k_{21}\left(t\right)\\ k_{21}\left(t\right) & k_{22}\left(t\right) \end{array}\right)\left(\begin{array}{cc} 0 & -a_{0}\\ 1 & -a_{1} \end{array}\right)\\ +\left(\begin{array}{cc} k_{11}\left(\tau -t\right) & k_{21}\left(\tau -t\right)\\ -k_{21}\left(\tau -t\right) & k_{22}\left(\tau -t\right) \end{array}\right)\left(\begin{array}{cc} 0 & b_{0}\\ 0 & b_{1} \end{array}\right), \end{array}$$

implying that

(64) $$k_{11}^{\prime }\left(t\right)=-k_{21}\left(t\right),\;k_{21}^{\prime }\left(t\right)=k_{22}\left(t\right)$$

and

(65) $$k_{22}^{\prime }\left(t\right)=-a_{0}k_{21}\left(t\right)-a_{1}k_{22}\left(t\right)-b_{0}k_{21}\left(\tau -t\right)+b_{1}k_{22}\left(\tau -t\right).$$

Using (52) we arrive at

$$k_{22}^{\prime \prime }\left(t\right)=-2a_{0}k_{21}^{\prime }\left(t\right)+\left(a_{0}^{2}-b_{0}^{2}\right)k_{11}\left(t\right)+\left(a_{1}^{2}-b_{1}^{2}\right)k_{22}\left(t\right)$$

and applying (64) we obtain the following system of second-order differential equations:

(66) $$\left\{\begin{array}{c} k_{11}^{\prime \prime }\left(t\right)=-k_{22}\left(t\right),\\ k_{22}^{\prime \prime }\left(t\right)=\left(a_{0}^{2}-b_{0}^{2}\right)k_{11}\left(t\right)+\left(a_{1}^{2}-b_{1}^{2}-2a_{0}\right)k_{22}\left(t\right). \end{array}\right.$$

The initial boundary conditions for this equation, based on (53), turn into

$$k_{11}^{\prime }\left(0\right)=0,\;k_{22}^{\prime }\left(0\right)=-\frac{\sigma^{2}}{2},$$

and

$$\left\{\begin{array}{c} k_{21}^{\prime }\left(0\right)=k_{22}\left(0\right),\\ -k_{21}^{\prime }\left(0\right)=-a_{0}k_{11}\left(0\right)+b_{0}k_{11}\left(\tau \right)+b_{1}k_{21}\left(\tau \right),\\ k_{22}^{\prime }\left(0\right)=-a_{1}k_{22}\left(0\right)-b_{0}k_{21}\left(\tau \right)+b_{1}k_{22}\left(\tau \right). \end{array}\right.$$

Combining them together with $k_{21}\left(\tau \right)=-k_{11}^{\prime }\left(\tau \right)$ by (64), we conclude that Equation (66) is supplemented with

(67) $$\left\{\begin{array}{c} a_{0}k_{11}\left(0\right)-k_{22}\left(0\right)-b_{0}k_{11}\left(\tau \right)+b_{1}k_{11}^{\prime }\left(\tau \right)=0,\\ a_{1}k_{22}\left(0\right)-b_{0}k_{11}^{\prime }\left(\tau \right)-b_{1}k_{22}\left(\tau \right)=\frac{\sigma^{2}}{2},\\ k_{11}^{\prime }\left(0\right)=0,\;k_{22}^{\prime }\left(0\right)=-\frac{\sigma^{2}}{2}. \end{array}\right.$$

Consequently, we may rewrite (66) as a second-order system of equations in the vector form

$$\left(\begin{array}{c} k_{11}^{\prime \prime }\left(t\right)\\ k_{22}^{\prime \prime }\left(t\right) \end{array}\right)=G\left(\begin{array}{c} k_{11}\left(t\right)\\ k_{22}\left(t\right) \end{array}\right)$$

with

(68) $$G=\left(\begin{array}{cc} 0 & -1\\ a_{0}^{2}-b_{0}^{2} & a_{1}^{2}-b_{1}^{2}-2a_{0} \end{array}\right).$$

The general solution of this equation is

(69) $$\left(\begin{array}{c} k_{11}\left(t\right)\\ k_{22}\left(t\right) \end{array}\right)=C\left(t\right)\left(\begin{array}{c} k_{11}\left(0\right)\\ k_{22}\left(0\right) \end{array}\right)+S\left(t\right)\left(\begin{array}{c} k_{11}^{\prime }\left(0\right)\\ k_{22}^{\prime }\left(0\right) \end{array}\right),$$

where

(70) $$C\left(t\right)=\sum_{k=0}^{\infty }\frac{t^{2k}}{(2k)!}G^{k}\;\mathrm{and}\;S\left(t\right)=\sum_{k=0}^{\infty }\frac{t^{2k+1}}{(2k+1)!}G^{k}.$$

Since $C^{\prime }\left(t\right)=GS\left(t\right)$ and $S^{\prime }\left(t\right)=C\left(t\right)$, we also have

(71) $$\left(\begin{array}{c} k_{11}^{\prime }\left(t\right)\\ k_{22}^{\prime }\left(t\right) \end{array}\right)=GS\left(t\right)\left(\begin{array}{c} k_{11}\left(0\right)\\ k_{22}\left(0\right) \end{array}\right)+C\left(t\right)\left(\begin{array}{c} k_{11}^{\prime }\left(0\right)\\ k_{22}^{\prime }\left(0\right) \end{array}\right).$$

We can rewrite (67) as the following equation:

(72) $$G_{1}\left(\begin{array}{c} k_{11}\left(0\right)\\ k_{22}\left(0\right) \end{array}\right)+G_{2}\left(\begin{array}{c} k_{11}\left(\tau \right)\\ k_{22}\left(\tau \right) \end{array}\right)+G_{3}\left(\begin{array}{c} k_{11}^{\prime }\left(\tau \right)\\ k_{22}^{\prime }\left(\tau \right) \end{array}\right)=\left(\begin{array}{c} 0\\ \frac{\sigma^{2}}{2} \end{array}\right),$$

where

(73) $$G_{1}=\left(\begin{array}{cc} a_{0} & -1\\ 0 & a_{1} \end{array}\right),\;G_{2}=\left(\begin{array}{cc} -b_{0} & 0\\ 0 & b_{1} \end{array}\right),\;G_{3}=\left(\begin{array}{cc} b_{1} & 0\\ -b_{0} & 0 \end{array}\right).$$

Using (69) and (71) for t=τ, we rewrite condition (72) as

$$\left(G_{1}+G_{2}C\left(\tau \right)+G_{3}GS\left(\tau \right)\right)\left(\begin{array}{c} k_{11}\left(0\right)\\ k_{22}\left(0\right) \end{array}\right)+\left(G_{2}S\left(\tau \right)+G_{3}C\left(\tau \right)\right)\left(\begin{array}{c} 0\\ -\frac{\sigma^{2}}{2} \end{array}\right)=\left(\begin{array}{c} 0\\ \frac{\sigma^{2}}{2} \end{array}\right).$$

Consequently, we obtain

(74) $$\left(\begin{array}{c} k_{11}\left(0\right)\\ k_{22}\left(0\right) \end{array}\right)={\left(G_{1}+G_{2}C\left(\tau \right)+G_{3}GS\left(\tau \right)\right)}^{-1}\left(I+G_{2}S\left(\tau \right)+G_{3}C\left(\tau \right)\right)\left(\begin{array}{c} 0\\ \frac{\sigma^{2}}{2} \end{array}\right)$$

and

$$\left(\begin{array}{c} k_{11}\left(t\right)\\ k_{22}\left(t\right) \end{array}\right)=C\left(t\right)\left(\begin{array}{c} k_{11}\left(0\right)\\ k_{22}\left(0\right) \end{array}\right)+S\left(t\right)\left(\begin{array}{c} 0\\ -\frac{\sigma^{2}}{2} \end{array}\right),\;t\in \left(0,\tau \right).$$

**Remark** **1.** *Our approach implies that the function $k_{22}$ satisfies, by (66), the following fourth-order differential equation*

(75) $$k_{22}^{\left(iv\right)}\left(t\right)-\left(a_{1}^{2}-b_{1}^{2}-2a_{0}\right)k_{22}^{\prime \prime }\left(t\right)+\left(a_{0}^{2}-b_{0}^{2}\right)k_{22}=0.$$

*If we go back to (64) and (65), and if we take m=1, $a_{1}=a$, $a_{0}=k$, $b_{1}=-b$, $b_{0}=0$, $k_{21}\left(t\right)=g\left(t\right)$, and $k_{22}\left(t\right)=C\left(t\right)$, then we get the same equation as Equation (10) in [29], where it is postulated that a general solution of Equation (10) is of the form*

$$C\left(t\right)=A_{c}\cos\left(\omega \left(t-\tau /2\right)\right)+A_{s}\sin\left(\omega \left(t-\tau /2\right)\right),$$

*with constants $A_{c}$, $A_{s}$, ω derived separately. However, observe that no matter what the constants are, we obtain*

$$C^{\prime \prime }\left(t\right)=-\omega^{2}C\left(t\right),$$

*but C(t) should be a general solution of (75). A similar approach was used in [31] (Appendix B) with Equation (B2), where $b_{1}=0$, $b_{0}=g/Q_{0}$, $a_{1}=1/Q_{0}$, $a_{0}=1$, and $k_{22}\left(t\right)=\phi_{vv}\left(t\right)$.*

#### 4.4.1. General Form of the Matrices C(t) and S(t) in (70)

To calculate the matrices C(t) and S(t), we find the eigenvalues of the matrix *G*. The characteristic equation det(zI−G)=0 for the matrix *G* in (68) is given by

$$z^{2}-\mathrm{tr}\left(G\right)z+\det\left(G\right)=0,$$

where

(76) $$\mathrm{tr}\left(G\right)=a_{1}^{2}-b_{1}^{2}-2a_{0}\;\mathrm{and}\;\det\left(G\right)=a_{0}^{2}-b_{0}^{2}.$$

Thus, we arrive at

(77) $$z^{2}-\left(a_{1}^{2}-b_{1}^{2}-2a_{0}\right)z+\left(a_{0}^{2}-b_{0}^{2}\right)=0,$$

whose roots are

(78) $$z_{\pm }=\frac{1}{2}\left(a_{1}^{2}-b_{1}^{2}-2a_{0}\pm \sqrt{\Delta }\right),$$

where

$$\Delta ={\left(a_{1}^{2}-b_{1}^{2}-2a_{0}\right)}^{2}-4\left(a_{0}^{2}-b_{0}^{2}\right).$$

Suppose that Δ>0, so that there are two different real eigenvalues. If detG<0 then $z_{+}z_{-}<0$ while if detG>0 then $z_{\pm }<0$ or $z_{\pm }>0$. The case of detG=0 implies that one of the roots is equal to 0. If Δ<0 then $z_{\pm }=\alpha e^{\pm i\beta }$, where α≥0 and β∈R, and we write $\sqrt{z_{\pm }}=\sqrt{\alpha }e^{\pm i\beta /2}$. Finally, if Δ=0 and detG≠0 then $z_{+}=z_{-}\neq 0$, while if detG=0 then $z_{+}=z_{-}=0$.

We have

(79) $$\begin{array}{c} C\left(t\right)=c_{0}\left(t\right)I+c_{1}\left(t\right)G,\;S\left(t\right)=s_{0}\left(t\right)I+s_{1}\left(t\right)G, \end{array}$$

for some functions $c_{i}$, $s_{i}$, i=0,1 that we now specify.

1. If Δ≠0 and det(G)≠0 then
  $$\begin{array}{c} c_{0}\left(t\right)=z_{+}c_{-}\left(t\right)-z_{-}c_{+}\left(t\right),\;c_{1}\left(t\right)=c_{+}\left(t\right)-c_{-}\left(t\right)\\ s_{0}\left(t\right)=z_{+}s_{-}\left(t\right)-z_{-}s_{+}\left(t\right),\;s_{1}\left(t\right)=s_{+}\left(t\right)-s_{-}\left(t\right) \end{array}$$
  where
  $$c_{\pm }\left(t\right)=\frac{\cosh(t\sqrt{z_{\pm }})}{z_{+}-z_{-}},\;s_{\pm }\left(t\right)=\frac{\sinh(t\sqrt{z_{\pm }})}{\left(z_{+}-z_{-}\right)\sqrt{z_{\pm }}}.$$
2. If Δ≠0 and det(G)=0 so that one of the roots is equal to zero, then $c_{0}$ and $c_{1}$ stay the same and
  $$s_{0}\left(t\right)=0,\;s_{1}\left(t\right)=\frac{\sinh(t\sqrt{z_{+}})}{z_{+}\sqrt{z_{+}}}.$$
3. Now suppose that Δ=0 and det(G)≠0, so that $z_{+}=z_{-}\neq 0$. Then
  $$c_{0}\left(t\right)=\cosh\left(t\sqrt{z_{+}}\right)-\frac{1}{2}\sqrt{z_{+}}t\sinh\left(t\sqrt{z_{+}}\right),\;c_{1}\left(t\right)=\frac{t}{2\sqrt{z_{+}}}\sinh\left(t\sqrt{z_{+}}\right)$$
  and
  $$\begin{array}{cc} s_{0}\left(t\right) & =\frac{3}{2\sqrt{z_{+}}}\sinh\left(t\sqrt{z_{+}}\right)-\frac{t}{2}\cosh\left(t\sqrt{z_{+}}\right),\\ s_{1}\left(t\right) & =\frac{t}{2z_{+}}\cosh\left(t\sqrt{z_{+}}\right)-\frac{1}{2z_{+}\sqrt{z_{+}}}\sinh\left(t\sqrt{z_{+}}\right). \end{array}$$
4. Finally, when Δ=0 and det(G)=0, then $z_{+}=z_{-}=0$ and we have
  $$c_{0}\left(t\right)=1,\;c_{1}\left(t\right)=\frac{1}{2}t^{2},\;s_{0}\left(t\right)=t,\;s_{1}\left(t\right)=\frac{1}{6}t^{3}.$$

#### 4.4.2. Stability Analysis of the Deterministic Delay Equation

To find the stability region for the second-order delay equation [39,40], substitute λ=iω with ω>0 into the characteristic Equation (58), separating it into real and imaginary parts:

$$a_{0}-\omega^{2}=b_{0}\cos\left(\omega \tau \right)+b_{1}\omega \sin\left(\omega \tau \right),\;a_{1}\omega =b_{1}\omega \cos\left(\omega \tau \right)-b_{0}\sin\left(\omega \tau \right),$$

and leading to a quartic equation for ω:

(80) $$\omega^{4}+\left(a_{1}^{2}-b_{1}^{2}-2a_{0}\right)\omega^{2}+\left(a_{0}^{2}-b_{0}^{2}\right)=0.$$

Now observe that if we substitute $z=-\omega^{2}$ into (77) then we obtain Equation (80). Consequently, solutions of (80) satisfy $\omega^{2}=-z_{\pm },$ where $z_{\pm }$ are as in (78). Thus, we can find a positive solution of (80) if and only if $z_{+}<0$ or $z_{-}<0$.

Here we classify the existence of these positive solutions in terms of det(G) and tr(G) as given in (76). Let *m* be the number of positive solutions of (80). Then

- m=0 iff det(G)>0 and $\mathrm{tr}\left(G\right)>-2\sqrt{\det(G)}$ or det(G)=0 and tr(G)≥0;
- m=1 iff det(G)<0 or det(G)=0 and tr(G)<0 or det(G)>0 and $\mathrm{tr}\left(G\right)=-2\sqrt{\det(G)}$;
- m=2 iff det(G)>0 and $\mathrm{tr}\left(G\right)<-2\sqrt{\det(G)}$.

Note that det(G)>0 iff $|a_{0}\left|>\right|b_{0}|$.

Recall that we assume $a_{0}>b_{0}$. Then $\alpha_{0}<0$ if and only if one of the following holds (see [39,40,41,42]):

1. $a_{1}>b_{1}$, m=0 and τ≥0;
2. $a_{1}>b_{1}$, m=1 and $\tau \in [0,\tau_{0}^{+})$ for some $\tau_{0}^{+}>0$;
3. $a_{1}>b_{1}$, m=2 and
  $$\tau \in \left[0,\tau_{0}^{+}\right)\cup \overset{k}{\underset{j=1}{⋃}}\left(\tau_{j-1}^{-},\tau_{j}^{+}\right),$$
  for some *k* and a sequence of delays $\tau_{j}^{\pm }$;
4. $a_{1}\leq b_{1}$, m=2 and
  $$\tau \in \overset{k}{\underset{j=0}{⋃}}\left(\tau_{j}^{-},\tau_{j}^{+}\right).$$

#### 4.4.3. Formulas for Variances

We need to find the inverse of the matrix $H:=G_{1}+G_{2}C\left(\tau \right)+G_{3}GS\left(\tau \right)$ and the second column of the matrix $I+G_{2}S\left(\tau \right)+G_{3}C\left(\tau \right)$. To this end, observe that

$$G_{1}+G_{2}C\left(\tau \right)+G_{3}GS\left(\tau \right)=G_{1}+c_{0}\left(\tau \right)G_{2}+c_{1}\left(\tau \right)G_{2}G+s_{0}\left(\tau \right)G_{3}G+s_{1}\left(\tau \right)G_{3}G^{2},$$

where $c_{i}=c_{i}\left(\tau \right)$, $s_{i}=s_{i}\left(\tau \right)$, i=0,1 are from (79) and the matrices $G_{1},G_{2},G_{3}$ are as in (73). We have

$$G_{2}G=\left(\begin{array}{cc} -b_{0} & 0\\ 0 & b_{1} \end{array}\right)\left(\begin{array}{cc} 0 & -1\\ \det(G) & \mathrm{tr}(G) \end{array}\right)=\left(\begin{array}{cc} 0 & b_{0}\\ b_{1}\det\left(G\right) & b_{1}\mathrm{tr}\left(G\right) \end{array}\right)$$

and

$$G_{3}G=\left(\begin{array}{cc} b_{1} & 0\\ -b_{0} & 0 \end{array}\right)G=\left(\begin{array}{cc} 0 & -b_{1}\\ 0 & b_{0} \end{array}\right),\;G_{3}G^{2}=\left(\begin{array}{cc} -b_{1}\det\left(G\right) & -b_{1}\mathrm{tr}\left(G\right)\\ b_{0}\det\left(G\right) & b_{0}\mathrm{tr}\left(G\right) \end{array}\right),$$

implying that the elements of the matrix $G_{1}+G_{2}C\left(\tau \right)+G_{3}GS\left(\tau \right)={\left(h_{ij}\left(t\right)\right)}_{i,j=1,2}$ are given by

(81) $$\begin{array}{ccc} h_{11} & =a_{0}-b_{0}c_{0}-b_{1}\det\left(G\right)s_{1},\; & h_{12}=-1+b_{0}c_{1}-b_{1}q_{1},\\ h_{21} & =\det\left(G\right)\left[b_{1}c_{1}+b_{0}s_{1}\right],\; & h_{22}=a_{1}+b_{1}q_{0}+b_{0}q_{1}, \end{array}$$

where

(82) $$q_{0}=c_{0}+\mathrm{tr}\left(G\right)c_{1},\;q_{1}=s_{0}+\mathrm{tr}\left(G\right)s_{1}.$$

The determinant of *H* is

$$\begin{array}{c} \det\left(H\right)=\left(a_{0}-b_{0}c_{0}-b_{1}\det\left(G\right)s_{1}\right)\left(a_{1}+b_{1}q_{0}+b_{0}q_{1}\right)\\ -\det\left(G\right)\left(b_{1}c_{1}+b_{0}s_{1}\right)\left(-1+b_{0}c_{1}-b_{1}q_{1}\right) \end{array}$$

which reduces to

(83) $$\begin{array}{c} \det\left(H\right)=a_{0}a_{1}+a_{0}\left(b_{1}q_{0}+b_{0}q_{1}\right)-a_{1}\left(b_{0}c_{0}+b_{1}\det\left(G\right)s_{1}\right)+\det\left(G\right)\left(b_{0}s_{1}+b_{1}c_{1}\right)\\ -\left(b_{0}c_{0}+b_{1}\det\left(G\right)s_{1}\right)\left(b_{1}q_{0}+b_{0}q_{1}\right)-\det\left(G\right)\left(b_{1}c_{1}+b_{0}s_{1}\right)\left(b_{0}c_{1}-b_{1}q_{1}\right). \end{array}$$

Next, we find the vector

$$\left(\begin{array}{c} h_{1}\\ h_{2} \end{array}\right)=\left(I+G_{2}S\left(\tau \right)+G_{3}C\left(\tau \right)\right)\left(\begin{array}{c} 0\\ 1 \end{array}\right).$$

We have

$$\begin{array}{c} I+G_{2}S\left(\tau \right)+G_{3}C\left(\tau \right)=I+s_{0}G_{2}+s_{1}G_{2}G+c_{0}G_{3}+c_{1}G_{3}G\\ =\left(\begin{array}{cc} 1-b_{0}s_{0}+b_{1}c_{0} & s_{1}b_{0}-b_{1}c_{1}\\ b_{1}\det\left(G\right)s_{1}-b_{0}c_{0} & 1+b_{1}s_{0}+b_{1}\mathrm{tr}\left(G\right)s_{1}+b_{0}c_{0} \end{array}\right) \end{array}$$

implying that

(84) $$h_{1}=b_{0}s_{1}-b_{1}c_{1},\;h_{2}=1+b_{0}c_{0}+b_{1}q_{1}.$$

Consequently, the diagonal matrix K(0) has on its diagonal by (74) the following values

(85) $$k_{11}\left(0\right)=\frac{\sigma^{2}}{2\det(H)}\left[h_{22}h_{1}-h_{12}h_{2}\right],\;k_{22}\left(0\right)=\frac{\sigma^{2}}{2\det(H)}\left[h_{11}h_{2}-h_{21}h_{1}\right],$$

where det(H) is as in (83), $h_{ij}$ as in (81) with $q_{i}$ in (82), and $h_{i}$ as in (84). We have

$$\begin{array}{c} h_{22}h_{1}-h_{12}h_{2}=\left(a_{1}+b_{1}q_{0}+b_{0}q_{1}\right)\left(b_{0}s_{1}-b_{1}c_{1}\right)-\left(-1+b_{0}c_{1}-b_{1}q_{1}\right)\left(1+b_{0}c_{0}+b_{1}q_{1}\right)\\ =1+a_{1}\left(b_{0}s_{1}-b_{1}c_{1}\right)+b_{0}\left(c_{0}-c_{1}\right)+b_{0}b_{1}\left(c_{0}q_{1}-q_{0}s_{1}\right)+b_{1}^{2}q_{0}c_{1}+b_{0}^{2}q_{1}s_{1}-b_{1}^{1}q_{1}^{2} \end{array}$$

and

$$\begin{array}{c} h_{11}h_{2}-h_{21}h_{1}=\left(a_{0}-b_{0}c_{0}-b_{1}\det\left(G\right)s_{1}\right)\left(1+b_{0}c_{0}+b_{1}q_{1}\right)\\ -\det\left(G\right)\left(b_{1}c_{1}+b_{0}s_{1}\right)\left(b_{0}s_{1}-b_{1}c_{1}\right). \end{array}$$

Note that if τ=0, $b_{0}=0,b_{1}=0$, then we have $\det\left(H\right)=a_{0}a_{1}$ and

$$k_{11}\left(0\right)=\frac{\sigma^{2}}{2a_{0}a_{1}},\;k_{22}\left(0\right)=\frac{\sigma^{2}}{2a_{1}},$$

that are the variances of the stationary solution of the noisy harmonic oscillator from Section 3.2 with $\beta =a_{0}$ and $\gamma =a_{1}$.

### 4.5. Is There a Fokker–Planck-like Equation for Differential Delay Equations?

We start with a few preliminaries with the ultimate goal of deriving a Fokker–Planck-like equation for the evolution of a ‘density’ under the action of noisy, delayed dynamics. The process ${\left\{x_{t}\right\}}_{t\geq 0}$ as in (27) defines a transition semigroup ${\left\{T^{t}\right\}}_{t\geq 0}$ by

$$T^{t}\psi \left(\phi \right)=E\left(\psi \left(x_{t}\right)|x_{0}=\phi \right),\;t\geq 0,\phi \in C,\psi \in B\left(C\right),$$

on the space B(C) of bounded Borel measurable functions ψ:C→R. For each t≥0 we define the Borel probability measure $\mu_{t}$ on the space *C* as the distribution of $x_{t}$, so that

$$\langle \psi ,\mu_{t}\rangle =\langle T^{t}\psi ,\mu_{0}\rangle ,\;t\geq 0,\psi \in B\left(C\right),$$

where we use the customary scalar product notation

$$\langle \psi ,\mu \rangle =\int_{C}\psi \left(\phi \right)\mu \left(d\phi \right).$$

Then ${\left\{\mu_{t}\right\}}_{t\geq 0}$ is said to solve the equation

(86) $$\frac{\partial }{\partial t}\mu_{t}=L^{*}\mu_{t},$$

where $L^{*}$ is a formal adjoint of the so-called *extended generator*L of the Markov process. The linear operator L:D→B(C) with domain D⊂B(C) is such that

$$\int_{0}^{t}\langle |L\psi |,\mu_{r}\rangle dr<\infty$$

and

$$\langle \psi ,\mu_{t}\rangle =\langle \psi ,\mu_{0}\rangle +\int_{0}^{t}\langle L\psi ,\mu_{r}\rangle dr$$

for all t>0 and ψ∈D.

Now, instead of dealing with the abstract Equation (86) we proceed as in [3,4,43]. We introduce a differential operator $L:C_{c}^{2}\left(R^{d}\right)\to B\left(C\right)$ defined by

$$Lg\left(\phi \right)=\sum_{i=1}^{d}F_{i}\left(\phi \right)\frac{\partial g}{\partial x_{i}}\left(\phi \left(0\right)\right)+\frac{1}{2}\sum_{i,j=1}^{d}{\left(\Sigma \Sigma^{T}\right)}_{ij}\left(\phi \right)\frac{\partial^{2}g}{\partial x_{i}\partial x_{j}}\left(\phi \left(0\right)\right),\;\phi \in C,$$

for $g\in C_{c}^{2}\left(R^{d}\right)$, where $C_{c}^{2}\left(R^{d}\right)$ is the space of functions with compact support that possess continuous derivatives up to second order. Here, ${\left[{\left(\Sigma \Sigma^{T}\right)}_{ij}\right]}_{i,j=1,\ldots ,d}$ is the matrix $\Sigma \Sigma^{T}$, where $\Sigma^{T}$ is the transpose of the matrix Σ. Consider the distribution μ(t) of $x\left(t\right)=x_{t}\left(0\right)$, t≥0, defined on $B(R^{d})$, the σ-algebra of Borel subsets of $R^{d}$, by

$$\mu \left(t\right)\left(B\right)=\mu_{t}\left\{\phi \in C:\phi \left(0\right)\in B\right\},\;B\in B\left(R^{d}\right).$$

Then ${\left\{\mu_{t}\right\}}_{t\geq 0}$ is said to solve the equation

(87) $$\frac{\partial }{\partial t}\mu \left(t\right)=L^{*}\mu_{t}$$

if

$$\int_{0}^{t}\langle |Lg|,\mu_{r}\rangle dr<\infty$$

and

(88) $$\int_{R^{d}}g\left(x\right)\mu \left(t\right)\left(dx\right)=\int_{R^{d}}g\left(x\right)\mu \left(0\right)\left(dx\right)+\int_{0}^{t}\langle Lg,\mu_{r}\rangle dr$$

for all t>0 and $g\in C_{c}^{2}\left(R^{d}\right)$. Note that we can represent the Borel measure μ(t) as $\mu \left(t\right)=\mu_{t}\circ \pi_{0}^{-1}$, where the projection map $\pi_{0}:C\to R^{d}$ is given by $\pi_{0}\left(\phi \right)=\phi \left(0\right)$, ϕ∈C. Thus, Equation (87) can be compared with (86) as it can be rewritten as

$$\frac{\partial }{\partial t}\mu_{t}\circ \pi_{0}^{-1}=L^{*}\mu_{t}.$$

Suppose that the drift coefficient *F* and the diffusion coefficient Σ in (26) are those with discrete delays

$$F\left(\phi \right)=F\left(\phi \left(\tau_{0}\right),\ldots ,\phi \left(\tau_{k}\right)\right),\;\Sigma \left(\phi \right)=\sigma \left(\phi \left(\tau_{0}\right),\ldots ,\phi \left(\tau_{k}\right)\right),$$

where k∈N, F and σ are given transformations on $R^{d(k+1)}$ and

$$0=\tau_{0}<\ldots <\tau_{k}=\tau .$$

We introduce the projection mapping $\pi_{\tau_{0},\ldots ,\tau_{k}}:C\to R^{d(k+1)}$

$$\pi_{\tau_{0},\ldots ,\tau_{k}}\left(\phi \right)=\left(\phi \left(-\tau_{0}\right),\ldots ,\phi \left(-\tau_{k}\right)\right),\;\phi \in C,$$

and the measure

$$\nu \left(t\right)\left(B\right)=\mu_{t}\circ \pi_{\tau_{0},\ldots ,\tau_{k}}^{-1}\left(B\right)=\mu_{t}\left\{\phi \in C:\pi_{\tau_{0},\ldots ,\tau_{k}}\left(\phi \right)\in B\right\},\;t\geq 0,B\in B\left(R^{d(k+1)}\right).$$

Observe that ν(t) is the distribution of $\left(x\left(t-\tau_{0}\right),\ldots ,x\left(t-\tau_{k}\right)\right)$. By using the change-of-variables formula we obtain

$$\langle Lg,\mu_{t}\rangle =\int_{R^{d}}\int_{R^{dk}}\left(\sum_{i=1}^{d}F_{i}\left(x,y\right)\frac{\partial g}{\partial x_{i}}\left(x\right)+\frac{1}{2}\sum_{i,j=1}^{d}a_{ij}\left(x,y\right)\frac{\partial^{2}g}{\partial x_{i}\partial x_{j}}\left(x\right)\right)\nu \left(t\right)\left(dx,y\right),$$

where $a_{ij}={\left(\sigma \sigma^{T}\right)}_{ij}$. Next, if $f_{\nu }$ is the density of the measure ν(t), assuming that it exists, with respect to the Lebesgue measure on $R^{d(1+k)}$, i.e., $\nu \left(t\right)\left(dx,dy\right)=f_{\nu }\left(x,y,t\right)dxdy$, then *f*, the density of x(t), exists and it is the marginal density obtained from $f_{\nu }$ by

$$f\left(x,t\right)=\int_{R^{dk}}f_{\nu }\left(x,y,t\right)dy.$$

Consequently, Equation (88) rewritten as

$$\begin{array}{cc} & \int_{R^{d}}g\left(x\right)f\left(x,t\right)dx=\int_{R^{d}}g\left(x\right)f\left(x,0\right)dx\\ & +\int_{0}^{t}\int_{R^{d}}\int_{R^{dk}}\left(\sum_{i=1}^{d}F_{i}\left(x,y\right)\frac{\partial g}{\partial x_{i}}\left(x\right)+\frac{1}{2}\sum_{i,j=1}^{d}a_{ij}\left(x,y\right)\frac{\partial^{2}g}{\partial x_{i}\partial x_{j}}\left(x\right)\right)f_{\nu }\left(x,y,r\right)dxdydr, \end{array}$$

is the weak form of the Fokker–Planck-type evolution equation

$$\frac{\partial }{\partial t}f\left(x,t\right)=-\sum_{i=1}^{d}\frac{\partial }{\partial x_{i}}\int_{R^{dk}}F_{i}\left(x,y\right)f_{\nu }\left(x,y,t\right)dy+\frac{1}{2}\sum_{i,j=1}^{d}\frac{\partial^{2}}{\partial x_{i}\partial x_{j}}\int_{R^{d}}a_{ij}\left(x,y\right)f_{\nu }\left(x,y,t\right)dy.$$

This is the central and desired result of this rather long and technical section.

## 5. Conclusions

It is clear from the above considerations that the dynamic origin of the (weak or strong) second law of thermodynamics is far from obvious. If the dynamics are given by typical (time-reversal-invariant) ordinary differential equations the behaviour of the Gibbs entropy is unclear (8) while in the same circumstance the conditional entropy is constant (10). The presence of noise does not alter the indeterminate behaviour of the Gibbs entropy but does induce a monotonic approach of the conditional entropy to its equilibrium value of zero (9).

The inclusion of temporal delays in the dynamics does not clarify things, but does increase the computational complexity of the situation. The Gibbs entropy might be monotone increasing (46) (strong form of the second law), whereas the conditional entropy may display a non-monotone approach (44) to equilibrium (47) (weak form of the second law).

We have emphasized the somewhat primitive state of the development of the underlying theory of the evolution of densities in infinite dimensional (delayed) dynamical systems, and why correction of this must necessarily precede a satisfactory understanding of the effects of delayed dynamics on entropy behaviours. There is much to be done!

### Why Bother?

One might wonder, given all of the technical problems involved in the treatment of density evolution in delayed systems, why is one bothering? The reason is straightforward. Biological processes are rife with delays modifying the dynamics, and models in many fields of the biological sciences contain significant delays at a variety of levels, e.g., ranging from gene regulation [44,45], through immunology [46], up to the cellular–clinical treatment level [47,48,49,50]. If we are ever to build a successful non-equilibrium thermodynamics for biological processes it will be essential to solve the problems pointed out in this study.

## Data Availability

No new data were created or analyzed in this study. Data sharing is not applicable to this article.
